# Evaluation of Health Management Information System data quality and information use for HIV/AIDS programs in public health facilities of Jimma town, Southwest Ethiopia

**DOI:** 10.1371/journal.pone.0357022

**Published:** 2026-09-21

**Authors:** Samson Tashome Abashu, Tilahun Fufa Debela, Muluembet Abera Wordofa, Berhane Megerssa Ereso

**Affiliations:** 1 Department of Health Policy and Management, Public Health Faculty, Institute of Health, Jimma University, Jimma, Ethiopia; 2 Department of Population and Family Health, Public Health Faculty, Institute of Health, Jimma University, Jimma, Ethiopia; Debre Tabor University, ETHIOPIA

## Abstract

**Background:**

Poor data quality is a critical challenge to meet the global and national health goals since performance of the health system cannot be adequately monitored. Despite significant investments, HIV/AIDS program data quality remains challenging in developing countries including Ethiopia to make informed decision. The aim of this study was to evaluate health management information system data quality and information use for HIV/AIDS program in Jimma town public health facilities, South West Ethiopia, 2022.

**Method:**

Facility-based cross-sectional evaluation with mixed methods was employed from June 22 to July 06/2022 at five public health facilities providing Anti-Retroviral Therapy in Jimma town. A total of 234 randomly selected HIV-positive patients’ medical records were reviewed, and a mobile-based data collection method was employed. Finally, resource availability, data quality and information use were analyzed separately and judged depending on judgment parameter.

**Results:**

The overall resource availability, data quality and information use were 75.1%, 77.2% and 70.4% respectively. None of the providers working on Voluntary Counselling and Testing service were trained on data quality. There was a lack of SmartCare, internet and printers in some health facilities. Report timeliness, data completeness and data consistency were 46.7%, 91.1% and 84.2% respectively. Performance monitoring team meetings were not held on a monthly basis and some facilities didn’t use the standard performance monitoring team logbook.

**Conclusion:**

The overall level of resource availability, data quality and information use were judged as good for each. However, report timeliness, data consistency and information uses were below the recommended national standards. Data quality related training should be provided for health care provides. SmartCare, internet, printers and guidelines should be supplied for the health facilities.

## 1. Introduction

Health Management Information System (HMIS) is the routine collection, aggregation, analysis, presentation and utilization of health and health related data for evidence based decisions for health workers, managers, policy makers and others [1,2]. Restructuring of health information systems has been a significant trend globally since the declaration of primary health care as an essential health care strategy at the Alma-Ata conference in 1978. As part of health system reform, developing countries also launched reforms to improve and expand health information systems [3]. However, studies in Sub-Saharan Africa (SSA) have revealed challenges with HMIS data quality, in terms of timeliness, completeness, consistency and poor utilization of HMIS tools [2,4,5].

Data quality is the degree to which a given dataset meets a user’s requirements and data are fit for their intended uses in operations, decision-making and planning. It is a prerequisite for ensuring HMIS information use [6]. On contrast, poor data quality is critical challenges to reaching the global and national health goals because the health system performance cannot be adequately monitored where data are of poor quality. Consequence of poor data quality includes everything from treatment quality to policy-level decision making. It leads to patient frustration and mistreatment, employee distrust of critical technology, decrease in efficiency and increase in bottlenecks, and poor and ineffective policy decisions [2,14].

Information use is the process through which decisions makers and stakeholders explicitly consider information in one or more steps of policy making, program planning and management, or service provision. Similarly, HIV/AIDS data is required at individual level, community level, facility level, and population level to provide individual level care and program level management [6,7].

Irrespective of huge supports by Non-Governmental Organizations and significant investments in technology, process development and human capital, HIV/AIDS program data quality remains challenging in developing countries to make informed decision [8]. In addition, the occurrence of COVID-19 pandemic has led to different challenges, and routine data accuracy check and RHIS performance review practices were also significantly reduced during the pandemic [9].

Study conducted in South Africa has revealed serious data-accuracy concerns in the routine data used to monitor PMTCT programs. This study indicated that an average accuracy between register and Routine Monthly Report was 51% and between the Routine Monthly Report and DHMIS database was 84% [10]. In addition, study conducted in Mozambique indicated that PMTCT data quality was 79% which is below expected national target. According to this study data consistency level between registration book and monthly reports was 89% and completeness of the data was 50% [11].

In Ethiopia, the electronics community HIV information system is not fully functional and is largely paper based system which does not have an effective grassroots structure. Assessment conducted at 47 health facilities in Addis Ababa indicated that less than 10% of the clients have valid residence information such as Kebele and mobile number recorded. Finding from routine RDQA by *Addis Ababa City Administration Health Bureau* in 16 health facilities showed over reporting in clients currently on treatment and testing positive. This assessment also showed underreporting on number of tests done and number of patients who are newly put on treatment [12]. In addition, study conducted in University of Gondar referral hospital indicates data completeness of ART clinic in both EMR and paper record is below national standard which is 76% and 78% respectively [13].

To solve HMIS data quality and information use problem, Ethiopian FMoH introduced information revolution and the government has been providing capacity building for health workforces [7]. Furthermore, revision has been made to HMIS and DHIS-2 was introduced. Similarly, SmartCare (EMR-ART) was also initiated to record and store HIV/AIDS data. Despite all this efforts, HIV AIDS data quality was not at the required level to make informed decision, provide quality care and support, and control the epidemic in Ethiopia [15,16]. To the best of our research, there is no published study conducted on HMIS or HIV AIDS program data quality and information use in Jimma town. The aim of this study was to identify the level of HIV/AIDS program data quality and information use in Jimma town public health facilities. Moreover, it was aimed to explore facilitators and barriers to HIV/AIDS data quality and information use.

## 2. Methods and materials

### 2.1 Study design, setting and period

Facility-based cross-sectional evaluation using mixed quantitative and qualitative methods was employed from June 22 to July 06/2022. The study was conducted in Jimma town which is located at 354 Km Southwest of Addis Ababa. There are 6 public health facilities (2 hospitals and 4 health centers) in the town of which five of them (two hospital and three health centers) are providing ART service. Jimma town health bureau reports of 2021 showed that there are 394 newly diagnosed HIV positive patients and a total of 5099 PLWHA in the town.

### 2.2 Population

Source populations were all HIV AIDS related documents and all HIV/AIDS program staffs in Jimma town public health facilities. Study populations were selected HIV AIDS program documents and purposively selected ART providers, data clerks, ART coordinators, Jimma town HIV/AIDS program coordinator and M and E officer.

### 2.3 Sample size determination and sampling procedures

A total of 234 randomly selected HIV-positive patients’ medical records were reviewed at five public health facilities providing ART service in the town. The sample size was determined by using single population proportion formula taking P 0.825 for overall average data quality of HMIS which was 82.5% from study conducted in Southern Ethiopia [17].


$$\begin{array}{c} n=\frac{((\text{Za}/\text{2})\text{2}\;)(\text{p})\;(\text{1}-\text{p})\;}{\text{d2}}=\text{\textasciitilde{}}\frac{(\text{1}.\text{96})\text{2}(\text{0}.\text{825})\;(.\text{175})\;}{(\text{0}.\text{0}\text{5})\text{2}},n=222 \end{array}$$

Since total population of patients on ART in the town is less than 10000 (i.e., 5099) correction formulas was used.

n=$\frac{no}{\text{1}+n\text{0}/N}$ =$\frac{\text{2}\text{2}\text{2}}{\text{1}+\text{2}\text{2}\text{2}/\text{5}\text{0}\text{9}\text{9}}$, $\frac{\text{2}\text{2}\text{2}}{\text{1}.\text{0}\text{4}}$ n = **213** and adding 10% for document which could be excluded (ineligible for document review) final sample size was **234**.

The total sample size was proportionally allocated to each public health facility based on number of patients on ART at each facility. Then patient charts were selected by systematic sampling technique using unique ART number. Similarly data on ART register was reviewed for selected patient charts. For qualitative study, a total of 17 purposively selected key informants were included in the study and sample size was guided by saturation of idea.

### 2.4 Eligibility criteria

Inclusion criteria were selected HIV/AIDS program documents which are readable and HIV/AIDS program staffs those worked at least for six months at the facility. Patient cards with omitted information, not readable and torn were excluded from the data collection.

### 2.5 Data collection tools and procedures

Data collection tools were adapted from standard guidelines and structured checklists were used for document review and recourse inventory [6,31]. In addition, semi-structured interview guide was developed depending on evaluation questions and used for the key informant interview. The tool was translated to local language (Afan Oromo and Amharic), and used for data collection after pretested at Agaro general hospital on 5% (equivalent to sample size = 12). A total of five data collectors were participated in data collection after taking two days training on evaluation objectives, data collection tools, mobile based data collection (ODK) and ethical issues. Trained data collectors were participated in pretest to adapt themselves to the tool for the final data collection and ODK application was employed. Key Informants were interviewed by principal evaluator after resource inventory and document review was conducted at the office of respective key informant. The whole procedure of data collection had been supervised and monitored to assure the data quality.

### 2.6 Data management and analysis

Quantitative data were downloaded from Kobotoolbox in excel form and exported into SPSS version 26 software for analysis. Then, descriptive statistics were calculated for variables and the results were presented by texts, tables and graphs. Field note and audio records were transcribed in to the same language and translated to English for further analysis. Translated data were coded, categorized and classified into themes. Then it was analyzed manually by thematic analysis technique and the results were narrated by triangulating with quantitative findings.

### 2.7 Ethical consideration

Ethical approval was obtained from the institutional review board of institute of health, Jimma University. Informed, voluntary, written and signed consent was obtained from each key informant. Name and other personal information that could compromise the respondents’ confidentiality were not recorded. In addition, face mask and sanitizer were availed for data collectors and key informants for covid-19 infection prevention.

### 2.8 Operational definitions

#### Availability.

In this evaluation, availability of resources required for HIV/AIDS program data quality and information use were assessed by ten indicators adapted from standard manuals.

#### Data quality.

Was evaluated by three major data quality dimensions namely; timeliness, completeness and consistency.

#### Timeliness.

Report timeliness was measured for six months routine HIV/AIDS report and said to be timely reported if submitted before 26th day of the reporting period. Accordingly, it was labeled as timeline if >90% of facilities report is submitted before the deadline.

#### Completeness.

It was measured by three indicators. Completeness of data on patient chart is the degree to which selected data is filled on sampled patient charts. Similarly, completeness of data on ART register is the degree to which selected variables are filled on ART register for sampled patient charts. In addition, completeness of data on report format is degree to which reportable data elements were filled against total number of reportable data elements supposed to be filled on HIV/AIDS program monthly reports. Overall completeness level was measured by relative weight given for the three indicators and judged against judgement parameter. Finally, facilities are expected to achieve data completeness level of >90% to be labeled as complete.

#### Consistency.

Data consistency was measured by four indicators. Consistency of data between patient chart and ART register was measured by assessing the level of data concordance for selected variables between the two recording tools. On the other hand, consistency over time was measured for twelve months HIV/AIDS monthly report and the report was labeled as inconsistent over time if it was increased or decreased by ≥ 33%. In addition, consistency between original record and reported data was measured by reviewing data on ART registration and HIV/AIDS monthly report. Overall, the health facilities’ data consistency level is said to be within the recommended national standard if it is  > 90%.

#### Information use.

It was measured by thirteen indicators and judged against agreed judgment parameter. The data were collected by reviewing PMT logbook and MDT minutes. In this evaluation PMT was said to be stablished as per standard if the facility head was chairperson of the PMT, HMIS focal person was secretary of the PMT and all case team coordinators were members of the PMT.

#### Judgment criteria.

Is a preset criteria developed by agreement with stakeholders and weight were given for each indicator by considering their relative importance in the evaluation. Accordingly, the cut of point was decided to be Very Good if ≥85%, Good if 70% – 84%, Fair if 55% – 69% and Critical if <55% [18].

## 3. Result

### 3.1 Description of the study participants

A total of 234 patient charts selected by systematic random sampling using unique ART number (143 from Jimma University medical center, 51 from Jimma health center, 27 from Shanen Gibe general hospital, 11 from Higher-2 health center, and 2 from Mandera Kochi health center) were reviewed. Similarly, data of sampled patient charts was reviewed on ART registers at respective health facilities. In addition, a total of 17 key informants (Jimma town HIV/AIDS program focal person, Jimma town M and E officer, ART focal person/coordinators, ART providers and data clerks) were included in the study.

### 3.2 Availability of resource

#### 3.2.1. Recording and reporting formats.

This study showed 90% overall availability of recording and reporting tools. There were no standard Performance monitoring team logbook and report timeliness and completeness tracking logbook in Jimma University Medical Center and Mandera Kochi health center. Similarly, Post-exposure prophylaxis register, multi-disciplinary team meeting minute and health institution report timeliness and completeness tracking logbook were not available in Higher-2 health center. On contrast, recording tools like ART register, PreP-register, PRE- ART register and VCT registers were available in all public health facilities. One respondent elaborated:

*“…there is no shortage of recording and reporting tool in our health facility. We have most report formats in soft copy and use it by printing it. In addition, recording tools like registers were provided by town health office for us”.* [A 34 years old male key informant]

#### 3.2.2 Guidelines.

According to this study, 55% of guidelines required for HIV/AIDS program data quality and information use were available. In Jimma health center, 75% of required guidelines were available and 50% of them were available in the other health facilities. All public health facilities have HMIS procedure and information use manual except Jimma health center. On contrary, Jimma health center is the only health facility which had HIV/AIDS data quality manual and HIV/AIDS information use manual. A respondent described this issue:

*“Even though different guidelines and manuals were provided from town health office, we do not have guidelines related to HIV/AIDS data quality in our health facility. This is due to our negligence since we didn’t request the office so far and even we can use it by downloading in soft copy from internet until we get it from the office…”* [A 30 years male key informant]

#### 3.2.3 Infrastructures.

According to this study, 80% of evaluated basic infrastructures were available in Jimma town public health facilities. All basic infrastructures were available in Jimma Medical center and Shanen Gibe hospital. On contrast, Mendera Kochi health center had 50% of the basic infrastructure during the study period. (Table 1)

**Table 1:** Availability of basic infrastructures in Jimma town public health facilities, 2022.

S.N	Variables	JUMC	SHGH	JHC	H2HC	MKHC
1	Availability of HMIS office or unit in the facility	Yes	Yes	Yes	Yes	Yes
2	Availability of continuous electricity supply	Yes	Yes	Yes	Yes	yes
3	Availability of desktop computer for HIV/AIDS data management	Yes	Yes	Yes	Yes	No
4	Availability of printer	Yes	Yes	Yes	No	No
5	Access to an internet network	Yes	Yes	No	No	No
6	Availability of functional telephone	Yes	Yes	Yes	No	yes
7	Availability of external hard drive	Yes	Yes	Yes	Yes	yes
8	Availability of ART-EMR	Yes	Yes	Yes	Yes	No
	Percentage	100%	100%	87.50%	62.50%	50%

One of the respondents elaborated the infrastructure problem:

*“....Internet service was interrupted for the last one month and we face problem to enter patient data on SmartCare. In addition, our facility enters monthly report on DHIS-2 at town health office due to internet interruption. Internet and telephone service interrupts frequently due to shortage of budget and providers were forced to use their mobile to trace lost to follow up.* [A 27 year old female key informant]

#### 3.2.4 Human resource.

According to this study, overall availability of trained human power on RHIS/HMIS data quality was 66.7%. All health facilities had assigned provider for VCT but none of them took data quality related training within the last six months. Unlike other health facilities, ART coordinators in Jimma University medical center and Shanen Gibe hospital took training on data quality since the last six months. A respondent described this issue:

*“…HIV/AIDS data quality is not achieved by only certain individuals and it needs involvement of all service delivery point provider. However, most providers were not trained on data quality since most of HIV/AIDS program related training is given for providers working on the program but not for other service delivery point provider”.* [A 28 years old male key informant]

### 3.3 Data quality

#### 3.3.1 Report timeliness.

From six months HMIS report, 46.67% of them were submitted to the next level according to the national schedule. Jimma health center, Higher-2 health center and Mendera Kochi health center had report timeliness level of 66.67%. On contrast, Shanen Gibe general hospital had report timeliness level of 33.3% and Jimma University medical center reported none of the six months report before the deadline. However, Most of the key informants stated that they submit monthly report before the deadline and report timeliness remains low due to edition of report on DHIS-2 after the deadline. A respondent clarified this issue:

*“Most of the time we submit monthly HIV/AIDS program reports before the deadline. However, timeliness level is recorded by DHIS-2 and it will automatically change the timelines level into zero when any data is entered or edited after the deadline…”* [A 29 years old data clerk]

#### 3.3.2 Completeness.

**3.3.2.1 Completeness of data on patient chart:** This study showed overall data completeness level of 96.2% (n = 234) on patient charts. Residence information like phone number, district and kebele were filled on 94%, 98.3% and 97.4% of sampled patient charts respectively. (Table 2)

**Table 2:** Data completeness level on patient charts in Jimma town public health facilities, 2022.

S.N	Variables	Completeness of patient chart by health facility					Total (n = 234)
		JUMC (n = 143)	SHGH (n = 27)	JHC (n = 51)	H2HC (n = 11)	MKHC (n = 2)	
	Phone number	95.8%	85.2%	92.2%	100%	100%	94%
	Age at entry	100%	92.6%	100%	100%	100%	99.1%
	ART start date	100%	100%	90.2%	100%	100%	97.9%
	Base line CD4 count (% if child)	98.6%	74.1%	96.1%	18.2%	50%	91%
	WHO clinical stage	100%	96.3%	98%	90.9%	100%	98.7%
	Region	95.8%	100%	100%	100%	100%	97.4%
	District	97.9%	100%	98%	100%	100%	98.3%
	Kebele	95.8%	100%	100%	100%	100%	97.4%
	Last follow up date	100%	96.3%	100%	100%	100%	99.6%
	ART regimen on last follow up	99.3%	96.3%	100%	72.7%	100%	97.9%
	Weight on last follow up	86%	92.6%	84.3	100%	100%	87.2%
	Overall completeness	97.2%	93.9%	96.26%	89.3%	95.45%	**96.2%**

Majority of key informants stated that there is a good attitude towards data completeness on patient charts like filling missed data and updating when patients come for follow up. A respondent elaborate this issue:

*“I register patient’s residential information and other required data on patient chart carefully when they start ART since it helps me to monitor patients response to ART and trace back once lost from follow up. I also fill data like phone number and address during follow up for patients previously not filled…”* [A 34 years old ART provider]

**3.3.2.2 Completeness of data on ART register:** This study revealed that overall data completeness level on ART register was 88.3% (n = 234). The highest level data completeness was observed on age at entry, ART start date and ART regimen on last follow up which was 99.6% for each variables. On contrast the least level of data completeness was observed on weight on last follow up (58.5%). Regarding data completeness on ART register by health facility, JUMC and Mendera MKHC had the highest level of data completeness which was 91.8% and 91.7% respectively.

Most of key informants stated that they give more attention in treating patients than filling data on ART register. The response of a 27 years old provider strengthens this concern as: *“Most of the time, I just focus on the assessment of the patient and managing the cases since there is high patient flow in our health facility. So, I prefer filling ART register when I am about to leave from the work and I suspect that this affect the completeness since I get tired …”*

**3.3.2.3 Completeness of data on report format:** According to this study, out of 510 data elements checked for report content completeness 445 (87.25%) of the data elements were completely filled in the report format. The highest level report completeness was observed at Jimma University Medical Center (92.16%) followed by Shanen Gibe Hospital (90.20%) and Mandera Kochi Health Center (88.24%). A respondent stated:

*“…I fill report format with zero if the service is not available or the result is not registered throughout the month. However, sometimes the report might be missed by mistake”.* [A 26 years old data clerk]

#### 3.3.3 Consistency.

**Fig 1:**
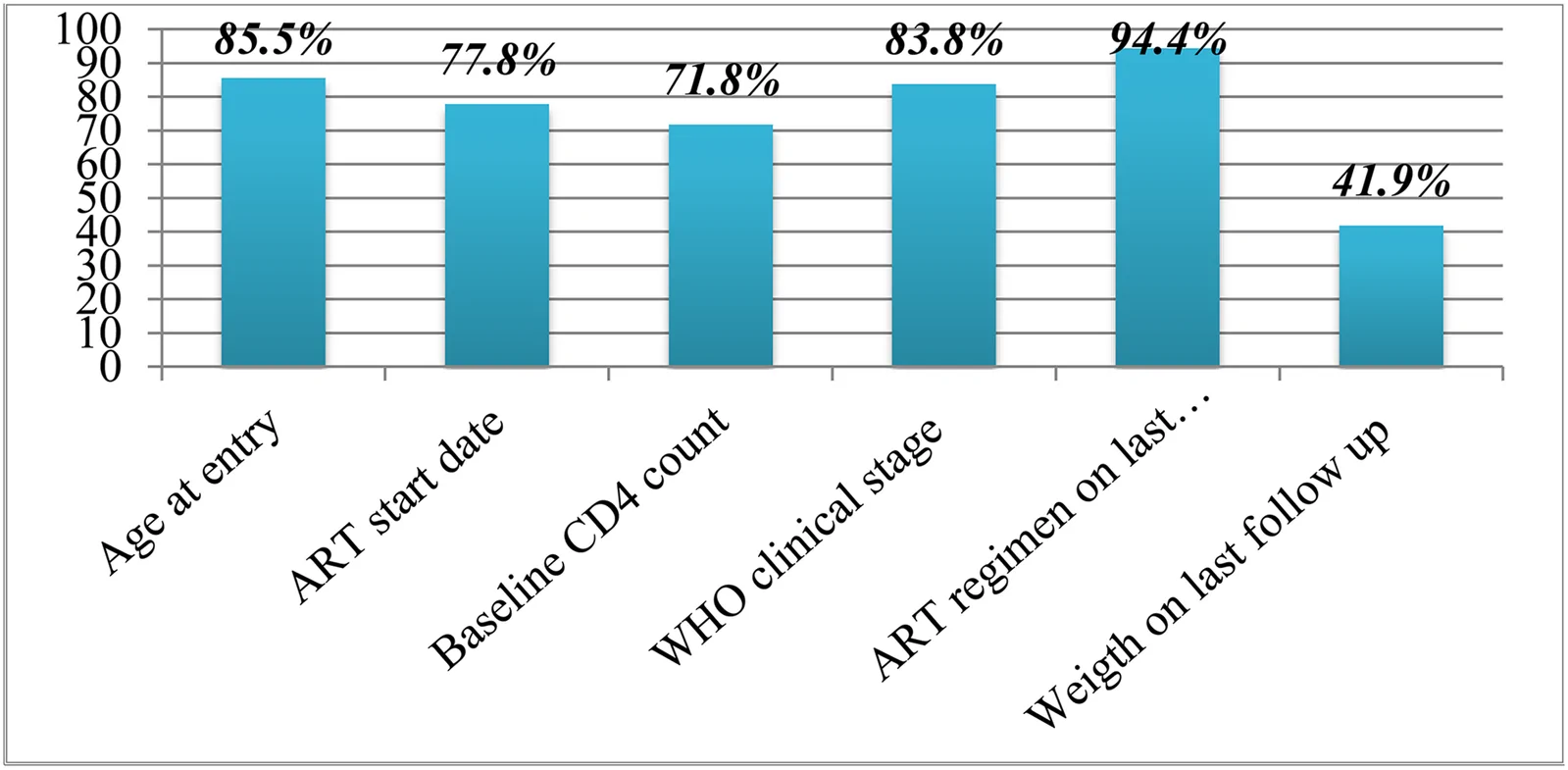
Level of data consistency between patient chart and ART register in Jimma town public health facilities, 2022.

**3.3.3.1 Consistency of data between patient chart and ART registration**: This study showed overall data consistency level of 75.85% between patient chart and ART register. The highest level of data consistency was observed on ART regimen on last follow up date (94.44%) and least level of consistency was observed for weight on last follow up date (41.88%) between patient chart and ART register (Fig 1).

Key informants identified two main reasons for data inconsistency, which includes filling of data by different person on patient chart and ART register, and lower attention for filling patient data as compared to treating clients. A participant elaborated this issue:

*“…. In our facility data on patient chart is filled by ART providers and data on ART register is filled by data clerk. In addition, Providers give more attention to service provision rather than data quality. They copy data on patient chart to registration when they are about to leave from work”* [26 years old data clerk]

**3.3.3.2 Consistency between reported data and data on ART register:** The overall data consistency between three months HIV/AIDS report and data on ART register was 105.8%. Three out of five health facilities had report consistency level find within acceptable range. On contrast, HIV/AIDS report was underreported in Jimma Health Center (134.76%) and Higher 2 Health center (113.56%). One participant clarified this issue:

*“Providers do not fill data on tally sheet properly which was recorded on register. In addition, report is not cross checked against data on tally sheet before report submission. This leads to false reporting which is over reporting or under reporting…”* [A 33 year old female KI]

**3.3.3.3 Consistency of HIV test report at VCT+PITC and HCT by population group:** According to this study, overall consistency of HIV Counselling and Testing (HCT) report by population group and PITC+VCT was 66.67%. It was 83.33% at Higher-2 health center and Mandera Kochi health center followed by Jimma health center (66.67%).

*“…HIV counseling and testing report is compiled from VCT and different service delivery point. providers give less attention in classifying tested clients into population group and this in turn leads to discrepancy between HCT by population group and VCT and PITC”.* [A 27 year old KI]

**3.3.3.4 Consistency over time:** This study revealed that eleven out of twelve months (91.7%) currently on ART report were consistent over time and report of March 2022 month was not consistent since it decreases from mean of the previous twelve months by 96.7% (Table 3). One participant described:

**Table 3:** Consistency over time of Currently on ART monthly report in Jimma town public health facilities, 2022.

S.N	Months	No of PLWHA currently on ART	Mean of currently on ART of the last 12 month	VF=$\frac{𝐂𝐮𝐫𝐫𝐞𝐧𝐭\;𝐨𝐧\;𝐀𝐑𝐓}{𝐌𝐞𝐚𝐧\;𝐨𝐟\;12\;𝐦𝐨𝐧𝐭𝐡𝐬\;}$	Consistency status
	April, 2021	4380	4304	1.0177	Yes
	May, 2021	4389	4288.5	1.0234	Yes
	June, 2021	4411	4323.1	1.0203	Yes
	July, 2021	4418	4399.3	1.0042	Yes
	August, 2021	4453	4377.7	1.0172	Yes
	September, 2021	4460	4353.8	1.0244	Yes
	October, 2021	4483	4371.3	1.0255	Yes
	November, 2021	4509	4389.7	1.0272	Yes
	December, 2021	4521	4408.7	1.0255	Yes
	January, 2022	4518	4427.1	1.0205	Yes
	February, 2022	4537	4443.3	1.0211	Yes
	March, 2022	4307	4453.7	**0.9671**	**No**
Overall consistency level					91.7%

*“…report of PLWHA currently on ART is filled with attention and we were asked for justification from the office if increased or decreased sharply. As a result, we checked and traced lost to follow up monthly, recorded transfer in properly and conduct death audit once death occurred”.* [A 34 years old male]

### 3.4 Information use

This study revealed overall information use level of 70.4% at facility level. All health facilities had established PMT as per standard and all HIV/AIDS case team prepared data visuals showing achievements toward targets. In addition, 73.3% of expected PMT was conducted over six months and 66.7% of conducted the PMT meeting was chaired by facility heads. On contrast, none of the conducted PMT was attended by head of town health office. Majority of key informants agreed that there is significant gap regarding information use at facility level and PMT meeting is conducted to fulfill the requirement. One participant stated:

*“It is difficult to get PMT members especially head of facility to conduct the meeting on regular basis. Sometimes, we conduct the meeting in the absence of facility head or with few members of the PMT for the requirement. Even there is a situation in which we fill on PMT logbook for one or two months without conducting the meeting…”* [A 28 year old male key informant]

Another participant added on this issue:

*“...PMT meeting is conducted to please higher level managers rather than solving problems. I think this is due to lack of awareness on PMT and information use as a whole”.* [A 28 year old female key informant]

### 3.5 Overall judgment

Based on the weight given for each dimension, the overall resource availability, data quality and information use were 75.1%, 77.2% and 70.4% respectively which scored good for each dimensions. (Table 4)

**Table 4:** Overall Judgment matrix for evaluation of HMIS data quality and information use in case of HIV/AIDS program in Jimma town public health facilities, 2022.

S.N	Dimension	Value given	Percentage achieved	Value achieved	Judgment criteria
1	Availability	100	75.1	**75.1**	Good
2	Data quality				
2.1	Timeliness	25	46.7	11.7	Good
2.2	Completeness	35	91.1	31.9	
2.3	Consistency	40	84.2	33.7	
	Total (for data quality)	100		**77.2**	
3	Information use	100	72.5	**72.5**	Good

## 4. Discussion

In this study, we identified the level of HIV/AIDS program data quality and information use in Jimma town public health facilities. In addition, we explored the facilitators and barriers to HIV/AIDS data quality and information use. Accordingly, the overall availability of recording and reporting tool was 90%. This finding is greater than the study conducted in Liberia and South West Shewa, Ethiopia with 55% and 25% respectively [19,20]. This difference might be due to difference in socioeconomic status of the country and the study setting respectively. The current study indicated that all health facilities had electric supply which is better than the study conducted in East Wollega (40%) and South West Shewa zone 77% [20,21]. The difference might be attributed by continuous electric supply in urban health facilities as compared to rural health facilities. In addition, four out of five (80%) of health facilities have ART-EMR (SmartCare) which is almost consistent with the study conducted in Mekelle town, Ethiopia with 83.3% EMR availability [22]. The consistency might be due to strategic focus of Ethiopian ministry of health in installing ART-EMR in high patient load facilities [23].

Our evaluation finding showed overall availability of trained human power on RHIS/HMIS data quality of 66.7%. This finding is better than the study conducted in East Wollega, Eastern Ethiopia and Southern nations and nationalities of Ethiopia which showed 36.6%, 52.7% and 52.2% of staffs took HIS training respectively [23–26]. The possible justification for this difference might be due to difference in sample size and study settings since urban health workers have greater opportunity for training.

This study revealed that 46.67% of six months reports were submitted to the next level according to the national schedule which was found to be far below the national expectation [16]. It was also lower than the study conducted in Harari region and Hadiya zone with report timeliness of 93.7% and 84% respectively [27,28]. This discrepancy might be attributed by data source of report timeliness since it was customized from DHIS-2 in current study.

On the other hand, the level of data completeness on patient chart was 96.2%.This finding is greater than the national minimum acceptable data completeness level and the study conducted in Addis Ababa which showed less than 10% of the clients on ART have filled valid residence information such as Kebele and mobile number [29]. The possible explanation for better achievement might be due to high staff commitment for data completeness on patient charts as indicated by qualitative findings. On contrast, data completeness on ART register was 88.3% and this finding is below nationally recommended level of data completeness [16].

Regarding report content completeness, 87.25% of the data elements were completely filled in the reporting format. This finding is comparable with recently conducted study in Hadiya Zone, Ethiopia (86%) and below the study conducted in Harari region, Ethiopia (93%) [27,28]. However, this result is greater than the study conducted in Southern Nations and Nationalities of Ethiopia with report content completeness of 83.3% [25]. The discrepancy might be attributed by improvements made to HIS throughout four years difference of study period like development of HIS guidelines, capacity building, and establishment and staffing of HIT structures at different levels [7].

This evaluation indicated data consistency between patient chart and ART register of 75.85% which is below nationally recommended level of data consistency. The possible justification for low achievement might be due to low level of staff attention towards data on ART register as indicated by qualitative findings. On the other hand, consistency of HIV/AIDS report over time was 91.7% which is greater than minimum expected level of data consistency. The possible justification for this level of achievement might be due to close follow up and attention to newly started ART and current on ART report by program coordinator.

The present evaluation finding revealed overall information use level of 70.4%. This finding was lower than the study conducted in North Gondar, Ethiopia which revealed 78.5% information utilization for decision making [30]. The possible explanation for lower level of information utilization might be due low staffs awareness towards information use, being busy of providers by other work and lower utilization of standard PMT logbook as elaborated by key informants.

## 5. Limitation of the evaluation

This evaluation might be susceptible to social desirability bias in which Key Informants might underreport undesirable attitudes or behaviors and over report more desirable attributes. Detail explanation was given for the key informants on purpose of the evaluation to minimize this bias. The other limitation might be the use of secondary data (document review) since there may be other important data that is not actually in the documents. To solve this problem, key informant interview was conducted to provide explanation for finding from the document review.

## 6. Conclusion

The overall level of resource availability, HIV/AIDS data quality and information use at facility level were judged as good for each dimension. However, there was a lack of infrastructure like functional printers, telephones and internet access. Majority of ART coordinators and VCT providers didn’t take data quality-related training or refreshment during the last six months. The level of data completeness was within recommended national standard. However, the level of report timeliness and data consistency were below recommended national standards. All health facilities had established PMT as per standard. However, PMT meeting, MDT meeting and LQAS were not held on regular basis. Thus, Jimma town health office should ensure the availability of basic resources to properly implement HMIS in the case of HIV AIDS program. Moreover, health facilities should strengthen PMT for improving data quality and information use.

## Supporting information

S1 Text Data collection tool, information sheet with consent form English and local languages versions. (PDF)

S2 Text STROBE checklist. (PDF)

S3 Text Data 1. (XLSX)

S4 Text Data 2. (XLSX)

## Abbreviations

CHIS: Community Health Information System
DHIS-2: District Health Information System-2
EMR: Electronic Medical Record
HCT: HIV Counseling and Testing
LQAS: Lot Quality Assurance Sampling
MDT: Multi-Disciplinary Team
PITC: Provided Initiated Testing and Counselling
PLHIVA: People living with HIV/AIDS
PMT: Performance Monitoring Team
