# Supplementary material for: Evaluation of Health Management Information System data quality and information use for HIV/AIDS programs in public health facilities of Jimma town, Southwest Ethiopia
Source: PLoS One. 2026 Sep 21;21(9):e0357022. doi: 10.1371/journal.pone.0357022 (PMC13592611; doi:10.1371/journal.pone.0357022)
Supplement: Supplementary file 1 [file pone.0357022.s001.pdf]

Annex 1: Data collection tools

Jimma University Institute of Health

Department of Health policy and Management, Health Monitoring and Evaluation Unit

Data collection tool for evaluation HMIS data quality and information use in case of HIV/AIDS program in Jimma town public health facilities, 2022.

Information sheet for hospital CEO/health center head

My name is _____________________and I am data collector assigned for the study conducted by Jimma university, health Monitoring and Evaluation post graduate student Samson Tashome on evaluation of HMIS data quality and information use in case of HIV/AIDS program.

Document review is one of data collection method used in this evaluation and I have planned to review patient chart, ART registration, HIV clinical care tally sheet, currently on ART and regimen tally sheet, PMT log book and report completeness and timeliness logbook. During the review, the confidentiality of the information will be kept in which the reviewed information will not specify names and other personal information's and the information will be utilized for evaluation purpose. The finding of this study will help to improve the quality of service provision.

May I continue to review the documents?

1. If **yes**, continue the review
2. If **no**, END

Part I: Documents Review checklist

Name of health facility ___________________________

1. **Timeliness**

Record review tool to assess report timeliness

Review the facilities data timeliness logbook or copy of HIV/AIDS report for the past six months (October 2021to March 2022) and check date of submission.<br>Hint: Select "**Yes"** if the report is submitted before or on the 26th day of the month, otherwise select "**No"**.		
**Months**	**Results**	
October 2021	1. Yes	2. No
November 2021	1. Yes	2. No
December 2021	1. Yes	2. No
January 2022	1. Yes	2. No
February 2022	1. Yes	2. No
March 2022	1. Yes	2. No

2. **Completeness**

A. Record review tool to assess data completeness on ART register

Review ART register and fill "yes" if selected variable is filled on register and "No" if selected variable is not filled on register			
**S.N**	**Variables**	**Results**	
1.	Age at entry	1. Yes	2. No
2.	Sex	1. Yes	2. No
3.	Kebele	1. Yes	2. No
4.	ART start date	1. Yes	2. No
5.	Weight	1. Yes	2. No
6.	Nutritional status screening result	1. Yes	2. No
7.	WHO clinical stage	1. Yes	2. No
8.	First line regimen	1. Yes	2. No
9.	CD4 count for adults & CD4% for children	1. Yes	2. No

B. Record review tool to assess completeness of data on report

Review six month (October 2021to March 2022) HIV/AIDS report for completeness of data on report format.<br>**Hint:** Count and record filled data elements (non-missing value) and missed value from total expected value in the table below.			
**Months**	**Results**		
	**Filled data elements**	**Missing value**	**% of completeness** $=(\frac{filled\ data}{expected\ data})*100$
October 2021			
November 2021			
December 2021			
January 2022			
February 2022			
March 2022			

3. <u>Consistency</u>

A. Record review tool to assess data consistency between patient chart and ART register

Check consistency of selected variables on patient chart and ART registers.<br>Hint: Select "Yes" if value recorded for selected variable on patient chart and ART register is concordant and select "No" if not.					
S.N	Variables	From patient chart	From registers	Consistency status	
1.	Age at entry			1. Yes	2. No
2.	Sex			1. Yes	2. No
3.	Region			1. Yes	2. No
4.	Woreda			1. Yes	2. No
5.	Kebele			1. Yes	2. No
6.	ART start date			1. Yes	2. No
7.	WHO clinical stage			1. Yes	2. No
8.	Last follow-up date			1. Yes	2. No
9.	Nutritional assessment result			1. Yes	2. No

B. Record review tool to assess HIV/AIDS data consistency over time

Review Jimma town health office monthly report of number of PLHIVs currently on ART for one year (April 2021 to March 2022).		
Months	number of PLHIVs currently on ART report	% of consistency $=(\frac{\text{No. of currently on ART at each month}}{total\ no.\ PLHIs\ over\ 1\ year})*100$
April, 2021		
May, 2021		
June, 2021		
July, 2021		
August, 2021		
September, 2021		
October, 2021		
November, 2021		
December, 2021		
January, 2022		
February, 2022		
March, 2022		
**Total**		

C. Record review tool to assess HIV/AIDS data consistency between ART register and report

Review and record the value on ART and post exposure prophylaxis (POP) register for selected indicators. Similarly, record the value on monthly report for three months (from January to March, 2022)

Indicators	January		February		March		**Total**		**VF=** $\left(\frac{Tot.on\,register}{Tot.on\,report}\right)*100$
	On register	On report	On register	On report	On register	On report	On register	On report	
Number of adults and children with HIV infection newly started on ART									
Number of patients enrolled in HIV care & treatment screened for TB									
Number of persons provided with post-exposure prophylaxis									
Number of undernourished PLWHIV who received therapeutic or food support									

D. Consistency between HIV test reports at VCT+PICT and HCT by population group

Review six month (October 2021to March 2022) HIV/AIDS report for clients receiving HIV test at VCT+PICT and reports of HCT by population group.			
**Months**	**Results**		
	Reports at VCT+PICT	HCT by population group	**DV=**
October 2021			
November 2021			
December 2021			
January 2022			
February 2022			
March 2022			

4. Information use

This check list is designed to assess information use at facility level. Review PMT log book and fill the check list.

1. Does the health facility have a performance monitoring or management team (PMT) in place?
   A. Yes
   B. No
2. How many times did the performance monitoring meetings take place during the past six months?
   A. Six times
   B. Five times
   C. Four times
   D. Three times
   E. Two times
   F. One times
   G. Not once
3. Does the health facility prepared data visuals (graphs, tables, maps, etc.) showing achievements toward targets
   A. Yes
   B. No
4. Were minutes of the performance monitoring/management meetings (PMT) maintained for the last three months?
   A. Yes available
   B. Not available
5. If yes, did they have discussions on RHIS management, like data quality, completeness, or timeliness of reporting (review PMT Meeting Minute book)?

6. If yes to Q≠5, have they made any decisions based on the discussions on RHIS related issues (review PMT Meeting Minute book)?

   A. Yes
   B. No

7. Did the head of the district health office attend any of the performance review management meetings?

   A. Yes
   B. No

Part II: Resource Inventory Checklist

This checklist will be used to conduct an inventory for availability of infrastructure and program resources for implementing HMIS in case of HIV/AIDS program in each health facilities. Please interview responsible body and check availability of resources to fill the table below.

Date of Assessment _______________

Name of health facility_________

S.N	Recording and reporting formats	Results	
1.	Is appointment card available?	1. Yes	2. No
2.	Is MPI available?	1. Yes	2. No
3.	Is PRE-ART Register available?	1. Yes	2. No
4.	Is ART Register available?	1. Yes	2. No
5.	Is PreP-register available?	1. Yes	2. No

6.	Is HIV clinical care tally sheet available?	1. Yes	2. No
7.	Is currently on ART and regimen tally sheet available?	1. Yes	2. No
8.	Is PEP register available?	1. Yes	2. No
9.	Is VCT Register available?	1. Yes	2. No
10.	Is hospital/health center/ service delivery report form available?	1. Yes	2. No
11.	Is copy of health facility's submitted monthly HIV/AIDS program reports available?	1. Yes	2. No
12.	Is health institution report timeliness and completeness tracking logbook available?	1. Yes	2. No
**Guidelines**			
13.	HIV/AIDS Data quality and information use manual	1. Yes	2. No
14.	Is HMIS indicator definition available?	1. Yes	2. No
15.	Is HMIS procedure manual and information use guideline available?	1. Yes	2. No
**Infrastructures**			
1.	Is there HMIS office or unit in the facility?	1. Yes	2. No
2.	Is there a continuous electricity supply?	1. Yes	2. No
3.	Does the health facility have desktop computer for HIV/AIDS data management?	1. Yes	2. No
4.	Is printer available?	1. Yes	2. No
5.	Is there access to an internet network?	1. Yes	2. No
6.	Is telephone available?	1. Yes	2. No
7.	Is external hard drive available?	1. Yes	2. No
8.	Is ART-EMR available?	1. Yes	2. No
**Human resource**			
1.	Does the health facility has HIT	1. Yes	2. No
2.	Does the health facility have data clerk responsible for entering data/compiling reports?	1. Yes	2. No
3.	If yes, did you take training related to data recording and/or data management within the last six month?	1. Yes	2. No

Part III: Key Informant Interview guide

Consent form for key informant interview

Hello, how are you? My name is ______________________and I am data collector assigned for the study Conducted by Jimma university, health Monitoring and Evaluation post graduate student Samson Tashome on evaluation of HMIS data quality and information use in case of HIV/AIDS program. The study is being conducted in Jimma town public health facilities and your facility is among selected health facilities to be included in the study. I will ask some questions and neither your name nor the names of any other respondent participating in this study will be included in the data set or in any report.

You may refuse to answer any question or choose to stop the interview at any time. However, I hope you will answer all of the questions, which will benefit the clients you serve and the nation. If there are questions that would be more accurately answered by someone better informed of any specifics I ask about, I would appreciate if you would introduce me to that person to help me collect any missing or incomplete information.

At this point, do you have any questions about the study? Do I have your agreement to proceed?

1. If **yes**, continue to the interview
2. If **no**, skip to the other participant

____ ______ / ______ / ______

Interviewer's signature indicating consent obtained Day Month Year

Start time _____________________________, End time ___________________

Supervisor's name _______________________, Signature ___________________

A. Interview guide for HIV/AIDS program coordinator/focal person

1. How do you see HIV/AIDS data quality in your facility, Do you think it is within recommended range*? (Prob: Do you submit HIV/AIDS report timely? If no why?)*
2. Do you think data on patient chart & registration is consistent with reported one? If no, why?
3. Do you think that data is filled on Register and reports completely? If no why?
4. Is there a system you are using to improve HIV/AIDS data quality? *(Prob: Do PMT stablished and being conducted monthly? If no why?)*
5. If yes to Q#4 what issues were discussed and action taken?
6. Do you think resources required to implement HMIS in case of HIV program are sufficient in your facility? *(Prob: Are infrastructure, human resource and recording tool sufficient? If not why? List missing)*

B. Interview guide for data clerks (HIT)

1. How do you see HIV/AIDS data quality in your facility, Do you think it is within recommended range*? (Prob: Do you submit HIV/AIDS report timely? If no why?)*
2. How do you check HIV/AIDS data quality?
3. How do you see the working environment where you are working? *(Prob: Are infrastructure, human resource and recording tool sufficient? If not why? List missing)*
4. What procedures are used in your facility to ensure data quality? *(Prob: Did you get feedback regarding data quality?)*

Thank you!

C. Interview guide for health care provider

1. How do thing resource availability for implementation of HMIS in terms of HIV program? *(Prob: like human resource, guidelines, registration and infrastructure)*
2. How do you perceive level of HI/AIDS data quality in your facility? (Prob: do you fill the register completely)
3. What methods do you use to assure HIV/AIDS data quality?
4. What problems you know that contribute to poor data quality?
5. Do you utilize the information you generate? If yes for what purpose?

Thank you!

Data Collection tool translated into local language (Afan Oromo)

Kutaa III: Unka walii galtee aaf-gaaffii gaafatamtoota ijoof (Afaan Oromootti kan hiikame)

Akkam jirta? Ani ______________________ kanin jedhamu sassaabduu odeeffannoo qo'annoo qulqullina sirna hooggansa odeeffannoo fayyaa dhibee HIV/Eedsii irratti Yuunivarsiitii Jimmaatti barataa digrii lammaa muummee hordoffiii fi gamaaggama fayyaa kan ta'e Saamsoon Tashoomeetiin hoojjetamaa jiruuti. qo'annoon kun dhaabbilee fayyaa mootummaa keessatti kan geggeeffamu yommuu ta'u dhaabbanni fayyaa keessanis kanneen qo'annoo kana keessatti hammataman keessaa isa tokko. Gaaffilee muraasasni ani si gaafadhu guutummmaa guutuutti yaadachuuf akka na gargaarutti deebiin ati deebistu ni waraabama. Maqaan kees ta'e kan nama biraa qo'annoo kana keessatti hirmaatuu gabaasa qo'annoo kanaa keessatti hin eeramu.
Qo'annoo kana keessatti hirmaachuuf akkasumas gaaffii kana gidduutti addaan kutuuf mirga guutuu qabda. Haa ta'uyyuu malee, deebiin ati kennitu maamiltoota kee waan fayyaduuf gaaffiiwwan kana guutummaatti akka deebistu abdii guutuun qaba. Gaaffiin ati namni biraa odeeffannoo dabalataa yookaan guutuu siif kenna jettee yaaddu yoo jiraate nama sana akkan gaafadhuuf akka wal na barsiiftu kabajaanin si gaafadha.

Hanga ammatti Gaaffii qabdaa? Aaf gaaffii kana itti fufuuf fedhii qabdaa?

1. Yoo tole ta'e, itti fufi.
2. Yoo miti ta'e, addaan kuti

______________________________________ ________ / ________ / ________

Mallattoo gaafatamaa heyyamamummaa isaa agarsiisu Guyyaa Ji'a Bara

Yeroo itti eegalame ______________________, Yeroo itti xumurame ____________________

Maqaa Suupparvaayizeraa _____________________, Mallattoo ____________________

A. Akeektuu aaf-gaaffii qindeessaa sagantaa dhibee HIV/AIDS'tiif

1. Sadarkaan qulqullina ragaalee dhibee HIV/AIDS akka dhaabbata fayyaa keessaniitti maal fakkaata? (*Dabalata: Gabaasa dhibee HIV/AIDS yeroodhaan ni ergituu? Yoo hin ergine maaliif?)*
2. Ragaan dhukkubsataa kaardii dhukkubsataa fi galmee irra jiru wal fakkaataadha jettee yaaddaa? Yoo hin taane maaliif?
3. Ragaan galmee fi gabaasa ji'aa irratti guutumaa guutuutti guutamaa jira jettee yaaddaa? Yoo hin taane maaliif?
4. Qulqullina ragaalee dhibee HIV/AIDS fooyyessuuf malootni isin fayyadamtan jiraa? (*Dabalata: Gareen hordoffii raawwii hudaa'ee jiraa? Ji'a ji'aan geggeeffamaa jiraa? Yoo geggeeffamaa hin jiraanne maaliif?)*
5. Yoo Gaaffii lakk-4'f deebiin kee eyyee ta'e, dhimmoota maalii fi tarkaanfii akkamiitu fudhatame? (*Dabalata: Hanqinoonni raawwii adda baafamaniiruu? Hanqinoota raawwiitiif sababni adda baafamaniiruu?)*
6. Qabeenyi sirna Odeeffannoo fayyaa geggeesssuuf barbaachisu ga'aadha jettee yaaddaa? (*Dabalata: Ijaarsi, humni namaa fi galmeewwan ga'aadha jettee yaaddaa? Kanneen hanqatan yoo jiraate tarreessi.)*

Galatoomi!

B. Akeektuu aaf-gaaffii data clerk'f yookiin HIT'tiif

1. Haala bakka hojii keetii akkamitti ibsita? (*Dabalata: Ijaarsi, humni namaa fi galmeewwan ga'aadha jettee yaaddaa? Kanneen hanqatan yoo jiraate tarreessi.)*
2. Sadarkaan qulqullina ragaalee dhibee HIV/AIDS akka dhaabbata fayyaa keessaniitti maal fakkaata? (*Dabalata: Gabaasa dhibee HIV/AIDS yeroodhaan ni ergituu? Yoo hin ergine maaliif?)*
3. Guutamiinsa ragaalee dhibee HIV/AIDS hoo akkamitti ilaalta? (*Dabalata: Ragaaleen dhibee HIV/AIDS kaardii dhukkubsataa fi galmee ART irra jiru guutummaa guutuutti ni guutammu?)*
4. Qulqullina ragaalee dhibee HIV/AIDS akkamitti mirkaneessitu?
5. Qulqullina ragaalee dhibee HIV/AIDS fooyyessuuf malootni isin fayyadamtan jiraa? (*Dabalata: Duub deebiin qulqullina ragaa HIV/AIDS irratti isiniif kennamee beekaa?)*

Galatoomi!

C. Akeektuu aaf-gaaffii ogeessa fayyaatiif

1. Qabeenyi sirna Odeeffannoo fayyaa geggeesssuuf barbaachisu ga'aadha jettee yaaddaa? (*Dabalata: Ijaarsi, humni namaa fi galmeewwan ga'aadha jettee yaaddaa? Kanneen hanqatan yoo jiraate tarreessi.)*
2. Sadarkaan qulqullina ragaalee dhibee HIV/AIDS akka dhaabbata fayyaa keessaniitti maal fakkaata? (*Dabalata: Ragaaleen dhibee HIV/AIDS kaardii dhukkubsataa fi galmee ART irra jiru guutummaa guutuutti ni guutammu?)*
3. Qulqullina ragaalee dhibee HIV/AIDS fooyyessuuf malootni isin fayyadamtan jiraa? (*Dabalata: barbaacha ijaanii ykn hordoffii ijaa ni taasistuu?)*
4. Qulqullina ragaalee gadaanaaf wantootni gumaachan maal fa'i jettee yaadda? *(Dabalata: Ji'oota ja'an darban keessatti leenjii qulqullina ragaalee fayyaan wal qabatu hirmaattee beekta?)*
5. Ragaalee oomishtan itti fayyadamaa jirtuu? Yoo ni fayyadamtu ta'e maal maal fa'iif itti fayyadamtu?

Galatoomi!

D. Akeektuu aaf-gaaffii qindeessaa dhibee HIV/AIDS magaalaa Jimmatiif

1. Akka dhaabbata fayyaa hawaasaa magaalaa Jimmaatti qabeenyi sirna Odeeffannoo fayyaa geggeesssuuf barbaachisu ga'aadha jettee yaaddaa? *(Dabalata: Ijaarsi, humni namaa fi galmeewwan ga'aadha jettee yaaddaa? Kanneen hanqatan yoo jiraate tarreessi.)*
2. Yoo deebiin kee lakk. 1f miti ta'e sababni/ootni maal fa'i?
3. Sadarkaan qulqullina ragaalee dhibee HIV/AIDS akka dhaabbata fayyaa keessaniitti maal fakkaata? (*Dabalata: Yeroon gabaasni itti ergamu, guutamiinsa ragaalee fi wal fakkatiinsi ragaalee reenjii gorfamu keessatti ni argamaa?)*
4. Qulqullina ragaalee gadaanaaf wantootni gumaachan maal fa'i jettee yaadda? (*Dabalata: leenjiin qulqullina ragaalee irratti ogeessota fayyaa fi kanneen biroof ni kennamaa? Yoo hin kennamu ta'e maaliif?*

Galatoomi!

Data Collection tool translated into local languages (Amharic)

ክፍል III: ወደ አማርኛ የተተረጎመው ቁልፍ መረጃ ሰጪ ቃለ መጠይቅ መመሪያ

ለቁልፍ መረጃ ሰጭ ቃለመጠይቅ የስምምነት ቅጽ

ሰላም እንደ ምን አለህ/ሽ? ስሜ ____________________ እባላለሁ። በጅማ ዩኒቨርሲቲ የጤና ክትትል እና ግምገማ ድህረ ምረቃ ተማሪ ለምካሄደው ጥናት መረጃ ሰብሳቢ ነኝ። ተማሪ ሳምሶን ተሾመ የጤና መረጃ አስተዳደር ሲስተም ጥራት ኤች አይቪ/ኤድስ ፕሮግራምን በተመለከተ የመረጃ ጥራት እና አጠቃቀም ላይ ጥናት እያካሀደ ነዉ። ጥናቱ በጅማ ከተማ የህዝብ ጤና ተቋማት እየተካሄደ ሲሆን በጥናቱ ውስጥ ከተካተቱት የጤና ተቋማት መካከል የእርስዎ ተቋም አንዱ ነው። አንዳንድ ጥያቄዎችን እጠይቅዎታለሁ እና ምላሽዎ ለመተንተን እንዲመች ይቀዳል፤፤ በዚህ ጥናት ውስጥ የእርስዎ ስምም ሆነ የሌላ ምላሽ ሰጪ ስም በመረጃ ስብስብ ውስጥ ወይም በማንኛውም ሪፖርት ውስጥ አይካተቱም።

በዚህ ቃለ መጠይቅ ውስጥ መሳተፍ በፈቃደኝነት ነው እና ማንኛውንም ጥያቄ ለመመለስ እምቢ ማለት ወይም በማንኛውም ጊዜ ቃለ መጠይቁን ለማቆም መምረጥ ይችላሉ። ነገር ግን፣ ሁሉንም ጥያቄዎች እንደሚመልሱ ተስፋ እያደረኩ ጥናቱ እርስዎ የሚያገለግሉትን ደንበኞች እና ሀገርን ይጠቅማል። ስለ ጠየቅኳቸው ማናቸውንም ዝርዝር ጉዳዮች በተሻለ በሚያውቅ ሰው፤ በበለጠ በትክክል የሚመልሱ ካሉ፣ የተሟላ መረጃ እንድሰበስብ እንዲረዳኝ ከዚያ ሰው ጋር ብታስተዋውቀኝ ደስ ይለኛል።

በዚህ ጊዜ ስለጥናቱ ምንም አይነት ጥያቄ አለዎት? ለመቀጠል የእርስዎ ስምምነት አለኝ?

1. አዎ ከሆነ፣ ቃለ መጠይቁን ይቀጥሉ

2. ካልሆነ፣ ወደ ሌላኛው ተሳታፊ ይዝለሉ

____ _____ ________ / ________ / የተገኘ

ስምምነትን የሚያመለክት የተሳታፊ ፊርማ ቀን ወር ዓ፡ም

የተጀመረበት ሰዓት _________, የተጠናቀቀነት ሰዓት ____________________

የተቆጣጣሪው ስም ________________________፣ፊርማ ________________

ሀ. የኤች አይቪ/ኤድስ ፕሮግራም አስተባባሪ የቃለ መጠይቅ መመሪያ

1. የኤች አይቪ/ኤድስ መረጃ የጥራት ደረጃን በእርስዎ ተቋም እንዴት ያዩታል፣ በሚመከረው ክልል ውስጥ ነው ብለው ያስባሉ? (ማብራርያ፡ የኤች አይቪ/ኤድስን ዘገባ በወቅቱ ታቀርባላችሁ? ካልሆነ ለምን?)
2. በታካሚ ካርድ እና መዝገብ ላይ ያለው መረጃ ከዘገበው ጋር የሚስማማ ይመስላችኋል? ካልሆነ ለምን?
3. መረጃ በመዝገብ እና በሪፖርት ላይ ሙሉ በሙሉ የምሞላ ይመስልዎታል? ካልሆነ ለምን?
4. የኤች አይቪ/ኤድስን መረጃ ጥራት ለማሻሻል እየተጠቀሙበት ያለው ሥርዓት አለ? (ማብራርያ፡ፒኤ**ምቲ (**PMT) ተቋቋመዉ በየወሩ እየተካሄደ ነው? ካልሆነ ለምን?)
5. ለጥያቄ ቁጥር 4 አዎ ከሆነ ምን ጉዳዮች ተወያይተዋል እና እርምጃ ተወስደዋል? (ምሳሌ፡- የአፈጻጸም ክፍተቶች ተለይተዋል? ዝቅተኛ አፈጻጸም ላላቸው ቁልፍ አመላካቾች የስር መንስኤ ትንተና ተካሂዷል?)
6. ኤችኤምአይኤስን ለመተግበር የሚያስፈልጉ ግብዓቶች በተቋሙ ውስጥ በቂ ናቸው ብለው ያስባሉ? (ማብራርያ፡ መሠረተልማት፣የሰው ኃይል እና የመመዝገቢያ መሳሪያዎች በቂና ቸው? ካልሆነ ለምን? የቀሩት ተንትን)

አመሰግናለሁ!

ለ. ለመረጃ ፀሐፊዎች ወይም ለኤች አይቲ የቃለ መጠይቅ መመሪያ

a. የሚሰሩበትን የስራ አካባቢ እንዴት ያዩታል? (ማብራርያ፡መሠረተ ልማት፣ የሰው ኃይል እና የመመዝገቢያ መሳሪያዎች በቂ ናቸው? ካልሆነ ለምን? የቀሩት ተንትን)

b. የኤች አይቪ/ኤድስን መረጃ ጥራት በተቋምዎ ውስጥ እንዴት ያዩታል፣ በሚመከረው ክልል ውስጥ ነው ብለው ያስባሉ? (ማብራርያ፡የኤች አይቪ/ኤድስን ዘገባ በወቅቱ ታቀርባላችሁ? ካልሆነ ለምን?)

c. የኤች አይቪ/ኤድስ መረጃ ሙሉነትስ እንዴት ያዩታል? (ማብራርያ፡ ሁሉም በART መዝገብ እና በታካሚ ቻርት ላይ ያሉት መረጃዎች ሙሉ በሙሉ ተሞልተዋል?)

d. የኤች አይቪ/ኤድስን መረጃ ጥራት እንዴት ነው የሚያረጋግጡት?

e. የኤች አይቪ/ኤድስን መረጃ ጥራት ለማረጋገጥ በእርስዎ ተቋም ውስጥ ምን ዓይነት ሂደቶች ጥቅም ላይ ይውላሉ? (ማብራርያ፡የውሂብ ጥራትን በተመለከተ ግብረ መልስ አግኝተዋል?)

አመሰግናለሁ!

ሐ. ለጤና እንክብካቤ ሰጪ የቃለ መጠይቅ መመሪያ

1. የኤች.ኤም.አይ.ኤስን ተግባራዊ ለማድረግ ከኤች አይቪ ፕሮግራም አንፃር የንብረት አቅርቦት እንዴት ይታያል? (ማብራርያ፡የሰው ሃብት፣መመሪያ፣መዝገብ እና መሠረተ ልማት በቂ ነዉ ብሎ ያስባሉ?፣የቀሩት ተንትን)
2. በእርስዎ ተቋም ውስጥ ያለውን የኤች አይአይ/ኤድስ መረጃ ጥራት ደረጃ እንዴት ይገነዘባሉ? በART መዝገብ እና በታካሚ ቻርት ላይ ያሉት መረጃዎች ሙሉ በሙሉ ተሞልተዋል?)
3. የኤች አይቪ/ኤድስን መረጃ ጥራት ለማረጋገጥ ምን አይነት ዘዴዎችን ትጠቀማለህ? (ማብራርያ፡የእይታ ቅኝት ዘዴ ትጠቀማለህ?)
4. ለደካማ የመረጃ ጥራት አስተዋጽኦ የሚያደርጉት ምን ችግሮች ያውቃሉ? (ማብራርያ፡ባለፉት ስድስት ወራት ውስጥ ከመረጃ ጥራት ጋር የተያያዘ ሥልጠና ወስደዋል?)
5. ያመነጬትን መረጃ ይጠቀማሉ? አዎ ከሆነ ለምን ዓላማ?

አመሰግናለሁ!

መ. የጅማ ከተማ ጤና ጽ/ቤት የኤች አይ ቪ/ኤድስ አስተባባሪ የቃለ መጠይቅ መመሪያ

6. በጅማ ከተማ የህብረተሰብ ጤና ተቋማት የኤች.ኤም.አይ.ኤስን ከኤች አይቪ ኘሮግራም አንፃር የግብዓት አቅርቦት እንዴት ይታያል? (ማብራርያ፡ የሰው ሃይል፣መመሪያ፣መዝገብ እና የመሰረተ ልማት አቅርቦት በቂ ናቸዉ? የቀሩትን ዘርዝር)
7. ለጥያቄ #1 በቂ ካልሆኑ ምክንያቱ ምንድን ነው?
8. በጅማ ከተማ የህዝብ ጤና ተቋማት የኤች አይቪ/ኤድስን መረጃ ጥራት እንዴት ያዩታል? (ማብራርያ፡ ከወቅታዊነት፣ምሉዕነት እና ከወጥነት አንፃር በሚመከረው ክልል ውስጥ ነው ብለው ያስባሉ? ካል ሆነ ምክንያቱ ምንድነዉ?
9. የኤችአይቪ/ኤድስ መረጃ አጠቃቀም ከሀገራዊ ግብ ጋር የሚሄድ ይመስላችኋል? (ማብራርያ፡ ፒ**ኤምቲ (**PMT) በየወሩ እየተካሄደ ነው?) ካል ሆነ ለምን?
10. ለዝቅተኛ የውሂብ ጥራት ምን አስተዋጽኦ ያደርጋል? (ማብራርያ፡ በኤችአይቪ/ኤድስ ፕሮግራም ላይ ለሚሠራ የጤና ባለሙያ ሥልጠና እየተሰጠ ነው? ካልሆነ ለምን?)

አመሰግናለሁ!
