# Supplementary material for: Evaluation of Health Management Information System data quality and information use for HIV/AIDS programs in public health facilities of Jimma town, Southwest Ethiopia
Source: PLoS One. 2026 Sep 21;21(9):e0357022. doi: 10.1371/journal.pone.0357022 (PMC13592611; doi:10.1371/journal.pone.0357022)
Supplement: Supplementary file 2 [file pone.0357022.s002.pdf]

STROBE Statement—Checklist of ***Evaluation of Health Management Information System data quality and information use; in case of HIV/AIDS program in Jimma town public health facilities, South West Ethiopia***

	Item No	Recommendation	Page No
**Title and abstract**	1	(*a*) Indicate the study's design with a commonly used term in the title or the abstract	**Page 2, line 30**
		(*b*) Provide in the abstract an informative and balanced summary of what was done and what was found	**Page 2, lines 30-40**
**Introduction**			
Background/rationale	2	Explain the scientific background and rationale for the investigation being reported	**Pages 3 to 4, lines 47 to 92**
Objectives	3	State specific objectives, including any prespecified hypotheses	**Page 4, lines 90-92**
**Methods**			
Study design	4	Present key elements of study design early in the paper	**Page 4, lines 95-96**
Setting	5	Describe the setting, locations, and relevant dates, including periods of recruitment, exposure, follow-up, and data collection	**Page 4, lines 95-96**
Participants	6	(*a*) Give the eligibility criteria, and the sources and methods of selection of participants	**Page 5, lines 111-103**
Variables	7	Clearly define all outcomes, exposures, predictors, potential confounders, and effect modifiers. Give diagnostic criteria, if applicable	**Page 6 and 7, lines 144-165**
Data sources/ measurement	8*	For each variable of interest, give sources of data and details of methods of assessment (measurement). Describe comparability of assessment methods if there is more than one group	***Page 5, lines 102-103*** ***Page 6, lines 144-165***
Bias	9	Describe any efforts to address potential sources of bias	**Page 16,lines 352-355**
Study size	10	Explain how the study size was arrived at	**Page 5, lines 105-117**
Quantitative variables	11	Explain how quantitative variables were handled in the analyses. If applicable, describe which groupings were chosen and why	**Page 6, lines 132-134**
Statistical methods	12	(*a*) Describe all statistical methods, including those used to control for confounding	**Page 6, lines 132-137**
		(*b*) Describe any methods used to examine subgroups and interactions	**NA**
		(*c*) Explain how missing data were addressed	**NA**
		(*d*) If applicable, describe analytical methods taking account of sampling strategy	**Page 5, lines 113-117**
		(*e*) Describe any sensitivity analyses	**NA**
**Results**			
Participants	13*	(a) Report numbers of individuals at each stage of study—eg numbers potentially eligible, examined for eligibility, confirmed eligible, included in the study, completing follow-up, and analysed	**Page 7, lines 168-173**
		(b) Give reasons for non-participation at each stage	**NA**

		(c) Consider use of a flow diagram	**NA**
Descriptive data	14*	(a) Give characteristics of study participants (eg demographic, clinical, social) and information on exposures and potential confounders	**Page 7, lines 168-173**
		(b) Indicate number of participants with missing data for each variable of interest	**NA**
Outcome data	15*	Report numbers of outcome events or summary measures	**Page 14 and 15, lines 308-311**
Main results	16	(*a*) Give unadjusted estimates and, if applicable, confounder-adjusted estimates and their precision (eg, 95% confidence interval). Make clear which confounders were adjusted for and why they were included	**NA**
		(*b*) Report category boundaries when continuous variables were categorized	**NA**
		(*c*) If relevant, consider translating estimates of relative risk into absolute risk for a meaningful time period	**NA**
Other analyses	17	Report other analyses done—eg analyses of subgroups and interactions, and sensitivity analyses	**NA**
**Discussion**			
Key results	18	Summarise key results with reference to study objectives	**Pages 14 and 15, lines 308-309**
Limitations	19	Discuss limitations of the study, taking into account sources of potential bias or imprecision. Discuss both direction and magnitude of any potential bias	**Page 17, lines 361-364**
Interpretation	20	Give a cautious overall interpretation of results considering objectives, limitations, multiplicity of analyses, results from similar studies, and other relevant evidence	**Pages 15 and 16, lines 314-361**
Generalisability	21	Discuss the generalisability (external validity) of the study results	**NA**
**Other information**			
Funding	22	Give the source of funding and the role of the funders for the present study and, if applicable, for the original study on which the present article is based	**Page 19, lines 406-408**
