# Supplementary material for: Evaluation of Health Management Information System data quality and information use for HIV/AIDS programs in public health facilities of Jimma town, Southwest Ethiopia
Source: PLoS One. 2026 Sep 21;21(9):e0357022. doi: 10.1371/journal.pone.0357022 (PMC13592611; doi:10.1371/journal.pone.0357022)
Supplement: Supplementary file 3 [file pone.0357022.s007.pdf]

Response to Reviewers

PONE-D-25-65187: Evaluation of Health Management Information System Data Quality and Information Use for HIV/AIDS Programs in Public Health Facilities of Jimma town, Southwest Ethiopia.

We sincerely appreciate your thoughtful feedback and are appreciative of the chance to submit the revised version of the manuscript. We have made changes to the manuscript in response to your insightful feedback, and we hope that the revised version of our manuscript will still be considered by PLOS ONE. Please see below is a point-by-point response to the comments raised by the editor and reviewers.

Kind regards,

S. No	Comments raised by reviewers	Authors' response
1.	The title is informative but grammatically awkward and overly long. The semicolon (";") is unnecessary and non-standard.	Comment accepted. We have rephrased the title and removed the semicolon on page 1, Line 1-3.
2.	Clarify the study design *(e.g.,* facility-based cross-sectional evaluation with mixed methods*).*	Comment accepted. We have modified the study design to "facility-based cross-sectional evaluation using mixed quantitative and qualitative methods" on page 2 line 28, and page 4, line 93.
3.	Explicitly state data quality dimensions and thresholds**.**	Comment accepted. We have explicitly stated expected national standards for each data quality dimensions on pages 6 and 7, lines 144-162.
4.	Avoid evaluative words like "good" without defining criteria.	Comment accepted. Judgement criteria is a preset criteria developed based on previous evidence and by agreement with stakeholders and weight was given for each indicator by considering their relative importance in the evaluation, and presented on page 7, line 167-169.
5.	Conceptual clarity of definitions of data quality and information use are provided but not clearly linked to the study framework.	Thank you. We have introduced the definition of data quality and information use as a whole since HIV/AIDS program is key component of health programs. In addition, we have highlighted HIV/AIDS program data quality on page 3, line 63-65.

6.	Reorganize the problem statement into Local evidence gap and study rationale and clearly state why Jimma town is an important setting**.**	We presented the problem statement from broader to specific (global to study area), and presented that there is no published study conducted on HMIS or HIV AIDS program data quality and information use in Jimma town on page 4, line 74-90.
7.	The term "facility based case study design" is methodologically ambiguous. So, clearly define the design, e.g.: "A facility-based cross-sectional evaluation using mixed quantitative and qualitative methods."	Comment accepted. We have modified the study design to "facility-based cross-sectional evaluation using mixed quantitative and qualitative methods" on page 2 line 29, and page 4, line 94.
8.	Justification for selecting 234 records is mathematically cluttered and hard to follow.	The sample size was determined by using single population proportion formula taking P 0.825 for overall average data quality of HMIS which was 82.5% from study conducted in Southern Ethiopia. Then, correction formula was used to get a sample size of 234 since the total population is less than 10,000 as presented on page 5, line 104-115.
9.	Inclusion of qualitative sample lacks clear rationale.	For qualitative study, purposive sampling technique was used in order to get information reach indviduals and sample size was determined by saturation of idea as stated 0n page 5, line 114-116. Accordingly, the purpose was key informants level of stake i.e being ART providers, ART focal person, M&E officer, ART coordinator and data clerk.
10.	Tools are mentioned but not sufficiently described. And it is unclear how qualitative data informed quantitative findings.	Qualitative and quantitative data are collected simultaneously, and qualitative results were narrated by triangulating with quantitative findings, page 6, lines 131-136
11.	Analytical methods are largely **descriptive**, yet the manuscript uses evaluative judgments ("good").	Mixed method was used in this evaluation, and a preset criteria judgment criteria which was developed by agreement with stakeholders was used and presented on page 7, line 168-170.
12.	Weighting system and judgment parameters are not clearly justified.	Comment accepted. We have included how judgement criteria were developed and weight was given for each indicator and presented on pages 6 and7, lines 145-170.
13.	Operational Definitions: Definitions are long, repetitive, and sometimes circular.	Comment accepted. We have modified the operational definitions and presented in informative way by including expected national standards for each data quality dimensions on pages 6 and 7 , lines 143-170.
14.	Results section includes interpretation that should be in Discussion.	In our evaluation qualitative results were presented by triangulating with quantitative findings to provide detail explanation for the quantitative results. Hence, we used introductory statement to present the actual responses of the

		key informants which in some ways resemble interpretation.
15.	Some percentages reported in text do not always align clearly with tables.	Comment accepted. Thank you so much for your careful review. The percentage in the narration (80%) indicates the overall availability of basic infrastructures in Jimma town public health facilities while the result in the table shows the availability of basic infrastructures in each health facilities. So, we have paraphrased the statement to make it informative on page 9, line 203&204.
16.	Use of terms such as overall, facility level, program level interchangeably.	Comment accepted. We have included your comments and removed inappropriate statement on page 10, line 225&226.
17.	Discussion largely repeats results**.**	We briefly narrated the main results under discussion section to compare our findings with different literatures and provide explanation.
18.	Limited critical reflection on methodological limitations.	Thank you. We have elaborated methodological limitations, and how the limitation could affect the interpretation on page 17, line 366 -370.
19.	Causal language is occasionally used despite descriptive design.	We provided the possible explanation for different level of achievement as compared to literature rather than cause effect relationship.
20.	Strengthen linkage to Ethiopian HMIS policy and HIV program goals.	Thank you. We discussed our findings with the expected national standards and presented by justifying with Ethiopian HMIS policy and HIV program goals on page 16, line 349-352.
21.	The limitations are acknowledged but **underdeveloped.** Please, clarify how these limitations affect interpretation.	Comment accepted. We have elaborated how the limitation could affect the interpretation on page 17, line 366-370.
22.	Conclusions align with objectives. However, some conclusions repeat numerical findings and recommendations are **generic**. I suggest you to tailor recommendations to specific bodies.	Comment accepted. We have incorporated your comments and forwarded the recommendation for specific bodies on page 17, line 378-380.
	**Response to editors queries**	
23.	Please clarify the source of funding (financial or material support) for your study. State what role the funders took in the study.	Comment accepted. The source of funding (support) and their role was stated on page 19, line 412-414.

24.	We note that you have provided funding information that is not currently declared in your Funding Statement. However, funding information should not appear in the Acknowledgments section or other areas of your manuscript.	We have removed from the acknowledgement section of the manuscript, and included the amended statements within the cover letter.
25.	Please amend your authorship list in your manuscript file to include the full name of author Tilahun Fufa Debela.	Thank you. We have included it.
26.	Your full ethics statement should only appear in the Methods section of your manuscript. If your ethics statement is written in any section besides the Methods, please delete it from any other section.	Comment accepted. We have deleted from other section of the manuscript other than method section.
27.	Please note that there are currently two tables labeled as "Table 1" on pages 9 and 13. Please renumber your tables and update the corresponding citations in the text to ensure they follow a sequential order.	Thank you. We have renumbered it.
28.	Please remove or anonymize all personal information (specific dates, health facility names, unique ART numbers), ensure that the data shared are in accordance with participant consent, and re-upload a fully anonymized data set. Please note that spreadsheet columns with personal information must be removed and not hidden as all hidden columns will appear in the published file.	We didn’t presented personal information like name of client or unique ART numbers in the manuscript, rather we assessed the completeness of patient card and registration by checking whether demographic information like name, age, unique ART numbers are completely filled or not.
