# Supplementary material for: Evaluation of Health Management Information System data quality and information use for HIV/AIDS programs in public health facilities of Jimma town, Southwest Ethiopia
Source: PLoS One. 2026 Sep 21;21(9):e0357022. doi: 10.1371/journal.pone.0357022 (PMC13592611; doi:10.1371/journal.pone.0357022)
Supplement: Supplementary file 4 [file pone.0357022.s005.docx]

**Evaluation of Health Management Information System data quality and information use; in case of HIV/AIDS program in Jimma town public health facilities, South West Ethiopia**

**1. General / Overall Comments**

- The topic is highly relevant to health systems strengthening, HIV program monitoring, and information use in low- and middle-income settings.
- The manuscript addresses an important evidence gap at sub-national level in Ethiopia and aligns well with PLOS ONE’s scope.
- The mixed-methods approach is appropriate; however, methodological clarity, analytical rigor, and narrative coherence need strengthening.
- Several sections suffer from repetition, grammatical issues, inconsistent terminology, and unclear judgment criteria.
- The manuscript would benefit from clearer linkage between objectives, methods, indicators, results, and conclusions.

**The title**

- The title is informative but grammatically awkward and overly long. The semicolon (“;”) is unnecessary and non-standard. I suggest the title to rephrased as “**Evaluation of Health Management Information System data quality and information use for HIV/AIDS programs in public health facilities of Jimma town, Southwest Ethiopia”**

**Abstract**

**Strengths**

- Includes background, methods, results, and conclusion.
- Provides key quantitative findings.

**Major Issues**

- Study design is described as a “facility based case study”*,* which is **unclear and potentially misleading.**
- The term “judged depending on judgment parameter” is vague.
- Results mix **resource availability, data quality, and information use** without clearly stating how each was operationalized.
- Conclusions repeat results without clear implications.

**I suggest to:**

- Clarify the study design *(e.g.,* facility-based cross-sectional evaluation with mixed methods*).*
- Explicitly state **data quality dimensions and thresholds.**
- Improve grammar and conciseness.
- Avoid evaluative words like “good” without defining criteria.

**Introduction**

**Strengths**

- Provides adequate background on HMIS and data quality challenges.
- Includes relevant international and national literature.
- Clearly identifies the **policy relevance** of HIV/AIDS data quality.
- **However,** the problem statement is diffuse because multiple issues (HMIS, HIV data, COVID-19, EMR, PMT) are introduced without a strong narrative flow.
- Conceptual clarity of definitions of data quality and information use are provided but not clearly linked to the study framework.
- The knowledge gap in Jimma town is mentioned late and could be emphasized earlier.
- Reorganize into: Local evidence gap and study rationale and clearly state **why Jimma town is an important setting.**

## Methods and Materials

### Regarding Study Design, the term “facility based case study design” is **methodologically ambiguous**.

### Clearly define the design, e.g.: “A facility-based cross-sectional evaluation using mixed quantitative and qualitative methods.”

### Study Population and Sampling: Justification for selecting **234 records** is mathematically cluttered and hard to follow.

### Inclusion of qualitative sample lacks clear rationale.

### Data Collection Tools and Procedures: Tools are mentioned but **not sufficiently described**. And it is unclear how qualitative data informed quantitative findings.

### Data Management and Analysis: Analytical methods are largely **descriptive**, yet the manuscript uses evaluative judgments (“good”).

### Weighting system and judgment parameters are not clearly justified.

### Operational Definitions: Definitions are long, repetitive, and sometimes circular.

## Results

### Major Issues

1. **Overly descriptive**
   - Results section includes interpretation that should be in Discussion.
2. **Inconsistencies**
   - Some percentages reported in text do not always align clearly with tables.
   - Use of terms such as overall*,* facility level*,* program level interchangeably.

**My Suggestions**

- Move interpretations to Discussion.
- Standardize terminology throughout.
- Renumber and simplify tables.
- Clearly label denominators and units.

## Discussion

### Strengths

- Findings are compared with national and international studies.
- Contextual explanations are provided.

### Major Issues

- Discussion largely **repeats results.**
- Limited critical reflection on **methodological limitations.**
- Causal language is occasionally used despite descriptive design.
- Avoid causal claims.
- Strengthen linkage to Ethiopian HMIS policy and HIV program goals.
- The limitations are acknowledged but **underdeveloped.** Please, clarify how these limitations affect interpretation.

## Conclusion and Recommendations

- Conclusions align with objectives. However, some conclusions repeat numerical findings and recommendations are **generic**. I suggest you to tailor recommendations to specific bodies.
