# Supplementary material for: Evaluation of Health Management Information System data quality and information use for HIV/AIDS programs in public health facilities of Jimma town, Southwest Ethiopia
Source: PLoS One. 2026 Sep 21;21(9):e0357022. doi: 10.1371/journal.pone.0357022 (PMC13592611; doi:10.1371/journal.pone.0357022)
Supplement: Peer review history

# Peer review: Decision Letter 0

**DOI:** 10.1371/journal.pone.0357022.r001

**Authors:** Worku Asferie

1 Jul 2026

Dear Dr. Ereso,

Thank you for submitting your manuscript to PLOS ONE. After careful consideration, we feel that it has merit but does not fully meet PLOS ONE’s publication criteria as it currently stands. Therefore, we invite you to submit a revised version of the manuscript that addresses the points raised during the review process.

Please submit your revised manuscript by Aug 15 2026 11:59PM. If you will need more time than this to complete your revisions, please reply to this message or contact the journal office at plosone@plos.org. When you're ready to submit your revision, log on to https://www.editorialmanager.com/pone/ and select the 'Submissions Needing Revision' folder to locate your manuscript file.

- A letter that responds to each point raised by the academic editor and reviewer(s). You should upload this letter as a separate file labeled 'Response to Reviewers'.
- A marked-up copy of your manuscript that highlights changes made to the original version. You should upload this as a separate file labeled 'Revised Manuscript with Track Changes'.
- An unmarked version of your revised paper without tracked changes. You should upload this as a separate file labeled 'Manuscript'.

If you would like to make changes to your financial disclosure, please include your updated statement in your cover letter. Guidelines for resubmitting your figure files are available below the reviewer comments at the end of this letter.

If applicable, we recommend that you deposit your laboratory protocols in protocols.io to enhance the reproducibility of your results. Protocols.io assigns your protocol its own identifier (DOI) so that it can be cited independently in the future. For instructions see: https://journals.plos.org/plosone/s/submission-guidelines#loc-laboratory-protocols. Additionally, PLOS ONE offers an option for publishing peer-reviewed Lab Protocol articles, which describe protocols hosted on protocols.io. Read more information on sharing protocols at https://plos.org/protocols?utm_medium=editorial-email&utm_source=authorletters&utm_campaign=protocols.

As the corresponding author, your ORCID iD is verified in the submission system and will appear in the published article. PLOS supports the use of ORCID, and we encourage all coauthors to register for an ORCID iD and use it as well. Please encourage your coauthors to verify their ORCID iD within the submission system before final acceptance, as unverified ORCID iDs will not appear in the published article. *Only*  the individual author can complete the verification step; PLOS staff *cannot*  verify ORCID iDs on behalf of authors.

We look forward to receiving your revised manuscript.

Kind regards,

Worku Necho Asferie, MSc

Academic Editor

PLOS One

Journal Requirements:

When submitting your revision, we need you to address these additional requirements.

1. Please ensure that your manuscript meets PLOS ONE's style requirements, including those for file naming. The PLOS ONE style templates can be found at

https://journals.plos.org/plosone/s/file?id=wjVg/PLOSOne_formatting_sample_main_body.pdf and https://journals.plos.org/plosone/s/file?id=ba62/PLOSOne_formatting_sample_title_authors_affiliations.pdf

2. We suggest you thoroughly copyedit your manuscript for language usage, spelling, and grammar. If you do not know anyone who can help you do this, you may wish to consider employing a professional scientific editing service.

The American Journal Experts (AJE) (https://www.aje.com/) is one such service that has extensive experience helping authors meet PLOS guidelines and can provide language editing, translation, manuscript formatting, and figure formatting to ensure your manuscript meets our submission guidelines. Please note that having the manuscript copyedited by AJE or any other editing services does not guarantee selection for peer review or acceptance for publication.

Upon resubmission, please provide the following:

- The name of the colleague or the details of the professional service that edited your manuscript

- A copy of your manuscript showing your changes by either highlighting them or using track changes (uploaded as a “Supporting Information” file)

- A clean copy of the edited manuscript (uploaded as the new *manuscript* file)

3. Thank you for stating the following financial disclosure:

[The author(s) received no specific funding for this work.].

At this time, please address the following queries:

a) Please clarify the sources of funding (financial or material support) for your study. List the grants or organizations that supported your study, including funding received from your institution.

b) State what role the funders took in the study. If the funders had no role in your study, please state: “The funders had no role in study design, data collection and analysis, decision to publish, or preparation of the manuscript.”

c) If any authors received a salary from any of your funders, please state which authors and which funders.

d) If you did not receive any funding for this study, please state: “The authors received no specific funding for this work.”

Please include your amended statements within your cover letter; we will change the online submission form on your behalf.

4. Thank you for stating the following in the Acknowledgments Section of your manuscript:

[The authors would like to thank Jimma University, Institute of Health, Department of Health Policy and Management for giving us this opportunity to conduct this scientific research. We are also thankful to JU-CBMP for financially supporting this research from Doris Duke Charitable Foundation fund. The mission of the Doris Duke Charitable Foundation is to improve the quality of people’s lives through grants supporting the performing arts, environmental conservation, medical research and child well-being, and through preservation of the cultural and environmental legacy of Doris Duke’s properties.]

We note that you have provided funding information that is not currently declared in your Funding Statement. However, funding information should not appear in the Acknowledgments section or other areas of your manuscript. We will only publish funding information present in the Funding Statement section of the online submission form.

Please remove any funding-related text from the manuscript and let us know how you would like to update your Funding Statement. Currently, your Funding Statement reads as follows:

[The author(s) received no specific funding for this work.]

Please include your amended statements within your cover letter; we will change the online submission form on your behalf.

5. Please amend your authorship list in your manuscript file to include the full name of author Tilahun Fufa Debela.

6. Your full ethics statement should only appear in the Methods section of your manuscript. If your ethics statement is written in any section besides the Methods, please delete it from any other section.

7. Please note that there are currently two tables labeled as "Table 1" on pages 9 and 13. Please renumber your tables and update the corresponding citations in the text to ensure they follow a sequential order.

8. We note that there is identifying data in the Supporting Information files <S3_Text_Data 1, S4_Text_Data 2>. Due to the inclusion of these potentially identifying data, we have removed this file from your file inventory. Prior to sharing human research participant data, authors should consult with an ethics committee to ensure data are shared in accordance with participant consent and all applicable local laws.

Data sharing should never compromise participant privacy. It is therefore not appropriate to publicly share personally identifiable data on human research participants. The following are examples of data that should not be shared:

-Name, initials, physical address

-Ages more specific than whole numbers

-Internet protocol (IP) address

-Specific dates (birth dates, death dates, examination dates, etc.)

-Contact information such as phone number or email address

-Location data

-ID numbers that seem specific (long numbers, include initials, titled “Hospital ID”) rather than random (small numbers in numerical order)

Data that are not directly identifying may also be inappropriate to share, as in combination they can become identifying. For example, data collected from a small group of participants, vulnerable populations, or private groups should not be shared if they involve indirect identifiers (such as sex, ethnicity, location, etc.) that may risk the identification of study participants.

Additional guidance on preparing raw data for publication can be found in our Data Policy (https://journals.plos.org/plosone/s/data-availability#loc-human-research-participant-data-and-other-sensitive-data) and in the following article: http://www.bmj.com/content/340/bmj.c181.long.

Please remove or anonymize all personal information (specific dates, health facility names, unique ART numbers), ensure that the data shared are in accordance with participant consent, and re-upload a fully anonymized data set. Please note that spreadsheet columns with personal information must be removed and not hidden as all hidden columns will appear in the published file.

If the reviewer comments include a recommendation to cite specific previously published works, please review and evaluate these publications to determine whether they are relevant and should be cited. There is no requirement to cite these works unless the editor has indicated otherwise.

Additional Editor Comments:

Editor Suggestion

Dear Authors,

Thank you for your submission. Here I attached the given comments and questions raised from the reviewers. Please incorporate the comments in the revised manuscript. Then, please attached both clean and track changed manuscript file during revised manuscript submission. Besides, focused on Plos One publication criteria before submission.

* Please Make the study Design "Facility based cross sectional with mixed methods"

From Reviewer 1

1. General / Overall Comments

� The topic is highly relevant to health systems strengthening, HIV program monitoring, and information use in low- and middle-income settings.

� The manuscript addresses an important evidence gap at sub-national level in Ethiopia and aligns well with PLOS ONE’s scope.

� The mixed-methods approach is appropriate; however, methodological clarity, analytical rigor, and narrative coherence need strengthening.

� Several sections suffer from repetition, grammatical issues, inconsistent terminology, and unclear judgment criteria.

� The manuscript would benefit from clearer linkage between objectives, methods, indicators, results, and conclusions.

The title

� The title is informative but grammatically awkward and overly long. The semicolon (“;”) is unnecessary and non-standard. I suggest the title to rephrased as “Evaluation of Health Management Information System data quality and information use for HIV/AIDS programs in public health facilities of Jimma town, Southwest Ethiopia”

Abstract

Strengths

� Includes background, methods, results, and conclusion.

� Provides key quantitative findings.

Major Issues

� Study design is described as a “facility based case study”, which is unclear and potentially misleading.

� The term “judged depending on judgment parameter” is vague.

� Results mix resource availability, data quality, and information use without clearly stating how each was operationalized.

� Conclusions repeat results without clear implications.

I suggest to:

� Clarify the study design (e.g., facility-based cross-sectional evaluation with mixed methods).

� Explicitly state data quality dimensions and thresholds.

� Improve grammar and conciseness.

� Avoid evaluative words like “good” without defining criteria.

Introduction

Strengths

• Provides adequate background on HMIS and data quality challenges.

• Includes relevant international and national literature.

• Clearly identifies the policy relevance of HIV/AIDS data quality.

� However, the problem statement is diffuse because multiple issues (HMIS, HIV data, COVID-19, EMR, PMT) are introduced without a strong narrative flow.

� Conceptual clarity of definitions of data quality and information use are provided but not clearly linked to the study framework.

� The knowledge gap in Jimma town is mentioned late and could be emphasized earlier.

� Reorganize into: Local evidence gap and study rationale and clearly state why Jimma town is an important setting.

Methods and Materials

• Regarding Study Design, the term “facility based case study design” is methodologically ambiguous.

• Clearly define the design, e.g.: “A facility-based cross-sectional evaluation using mixed quantitative and qualitative methods.”

• Study Population and Sampling: Justification for selecting 234 records is mathematically cluttered and hard to follow.

• Inclusion of qualitative sample lacks clear rationale.

• Data Collection Tools and Procedures: Tools are mentioned but not sufficiently described. And it is unclear how qualitative data informed quantitative findings.

• Data Management and Analysis: Analytical methods are largely descriptive, yet the manuscript uses evaluative judgments (“good”).

• Weighting system and judgment parameters are not clearly justified.

• Operational Definitions: Definitions are long, repetitive, and sometimes circular.

Results

Major Issues

1. Overly descriptive

o Results section includes interpretation that should be in Discussion.

2. Inconsistencies

o Some percentages reported in text do not always align clearly with tables.

o Use of terms such as overall, facility level, program level interchangeably.

My Suggestions

• Move interpretations to Discussion.

• Standardize terminology throughout.

• Renumber and simplify tables.

• Clearly label denominators and units.

Discussion

Strengths

• Findings are compared with national and international studies.

• Contextual explanations are provided.

Major Issues

• Discussion largely repeats results.

• Limited critical reflection on methodological limitations.

• Causal language is occasionally used despite descriptive design.

• Avoid causal claims.

• Strengthen linkage to Ethiopian HMIS policy and HIV program goals.

• The limitations are acknowledged but underdeveloped. Please, clarify how these limitations affect interpretation.

Conclusion and Recommendations

• Conclusions align with objectives. However, some conclusions repeat numerical findings and recommendations are generic. I suggest you to tailor recommendations to specific bodies.

[Note: HTML markup is below. Please do not edit.]

Reviewers' comments:

Reviewer's Responses to Questions

**Comments to the Author**

1. Is the manuscript technically sound, and do the data support the conclusions?

Reviewer #1: Yes

**********

2. Has the statistical analysis been performed appropriately and rigorously?

Reviewer #1: Yes

**********

3. Have the authors made all data underlying the findings in their manuscript fully available?

The PLOS Data policy

Reviewer #1: Yes

**********

4. Is the manuscript presented in an intelligible fashion and written in standard English?

Reviewer #1: No

**********

Reviewer #1: Evaluation of Health Management Information System data quality and information use; in case of HIV/AIDS program in Jimma town public health facilities, South West Ethiopia

1. General / Overall Comments

� The topic is highly relevant to health systems strengthening, HIV program monitoring, and information use in low- and middle-income settings.

� The manuscript addresses an important evidence gap at sub-national level in Ethiopia and aligns well with PLOS ONE’s scope.

� The mixed-methods approach is appropriate; however, methodological clarity, analytical rigor, and narrative coherence need strengthening.

� Several sections suffer from repetition, grammatical issues, inconsistent terminology, and unclear judgment criteria.

� The manuscript would benefit from clearer linkage between objectives, methods, indicators, results, and conclusions.

The title

� The title is informative but grammatically awkward and overly long. The semicolon (“;”) is unnecessary and non-standard. I suggest the title to rephrased as “Evaluation of Health Management Information System data quality and information use for HIV/AIDS programs in public health facilities of Jimma town, Southwest Ethiopia”

Abstract

Strengths

� Includes background, methods, results, and conclusion.

� Provides key quantitative findings.

Major Issues

� Study design is described as a “facility based case study”, which is unclear and potentially misleading.

� The term “judged depending on judgment parameter” is vague.

� Results mix resource availability, data quality, and information use without clearly stating how each was operationalized.

� Conclusions repeat results without clear implications.

I suggest to:

� Clarify the study design (e.g., facility-based cross-sectional evaluation with mixed methods).

� Explicitly state data quality dimensions and thresholds.

� Improve grammar and conciseness.

� Avoid evaluative words like “good” without defining criteria.

Introduction

Strengths

• Provides adequate background on HMIS and data quality challenges.

• Includes relevant international and national literature.

• Clearly identifies the policy relevance of HIV/AIDS data quality.

� However, the problem statement is diffuse because multiple issues (HMIS, HIV data, COVID-19, EMR, PMT) are introduced without a strong narrative flow.

� Conceptual clarity of definitions of data quality and information use are provided but not clearly linked to the study framework.

� The knowledge gap in Jimma town is mentioned late and could be emphasized earlier.

� Reorganize into: Local evidence gap and study rationale and clearly state why Jimma town is an important setting.

Methods and Materials

• Regarding Study Design, the term “facility based case study design” is methodologically ambiguous.

• Clearly define the design, e.g.: “A facility-based cross-sectional evaluation using mixed quantitative and qualitative methods.”

• Study Population and Sampling: Justification for selecting 234 records is mathematically cluttered and hard to follow.

• Inclusion of qualitative sample lacks clear rationale.

• Data Collection Tools and Procedures: Tools are mentioned but not sufficiently described. And it is unclear how qualitative data informed quantitative findings.

• Data Management and Analysis: Analytical methods are largely descriptive, yet the manuscript uses evaluative judgments (“good”).

• Weighting system and judgment parameters are not clearly justified.

• Operational Definitions: Definitions are long, repetitive, and sometimes circular.

Results

Major Issues

1. Overly descriptive

o Results section includes interpretation that should be in Discussion.

2. Inconsistencies

o Some percentages reported in text do not always align clearly with tables.

o Use of terms such as overall, facility level, program level interchangeably.

My Suggestions

• Move interpretations to Discussion.

• Standardize terminology throughout.

• Renumber and simplify tables.

• Clearly label denominators and units.

Discussion

Strengths

• Findings are compared with national and international studies.

• Contextual explanations are provided.

Major Issues

• Discussion largely repeats results.

• Limited critical reflection on methodological limitations.

• Causal language is occasionally used despite descriptive design.

• Avoid causal claims.

• Strengthen linkage to Ethiopian HMIS policy and HIV program goals.

• The limitations are acknowledged but underdeveloped. Please, clarify how these limitations affect interpretation.

Conclusion and Recommendations

• Conclusions align with objectives. However, some conclusions repeat numerical findings and recommendations are generic. I suggest you to tailor recommendations to specific bodies.

**********

what does this mean?). If published, this will include your full peer review and any attached files.

If you choose “no”, your identity will remain anonymous but your review may still be made public.

**Do you want your identity to be public for this peer review?**  For information about this choice, including consent withdrawal, please see our Privacy Policy

Reviewer #1: No

**********

[NOTE: If reviewer comments were submitted as an attachment file, they will be attached to this email and accessible via the submission site. Please log into your account, locate the manuscript record, and check for the action link "View Attachments". If this link does not appear, there are no attachment files.]

To ensure your figures meet our technical requirements, please review our figure guidelines: https://journals.plos.org/plosone/s/figures

You may also use PLOS’s free figure tool, NAAS, to help you prepare publication quality figures: https://journals.plos.org/plosone/s/figures#loc-tools-for-figure-preparation.

NAAS will assess whether your figures meet our technical requirements by comparing each figure against our figure specifications.

Attachment Submitted filename: Plose one reviewer comment.docx

# Author comment: Author response to Decision Letter 1

**DOI:** 10.1371/journal.pone.0357022.r002

21 Jul 2026

Response to Reviewers

PONE-D-25-65187: Evaluation of Health Management Information System Data Quality and Information Use for HIV/AIDS Programs in Public Health Facilities of Jimma town, Southwest Ethiopia.

We sincerely appreciate your thoughtful feedback and are appreciative of the chance to submit the revised version of the manuscript. We have made changes to the manuscript in response to your insightful feedback, and we hope that the revised version of our manuscript will still be considered by PLOS ONE. Please see below is a point-by-point response to the comments raised by the editor and reviewers.

Kind regards,

S. No Comments raised by reviewers Authors’ response

1. The title is informative but grammatically awkward and overly long. The semicolon (“;”) is unnecessary and non-standard. Comment accepted. We have rephrased the title and removed the semicolon on page 1, Line 1-3.

2. Clarify the study design (e.g., facility-based cross-sectional evaluation with mixed methods). Comment accepted. We have modified the study design to “facility-based cross-sectional evaluation using mixed quantitative and qualitative methods” on page 2 line 28, and page 4, line 93.

3. Explicitly state data quality dimensions and thresholds. Comment accepted. We have explicitly stated expected national standards for each data quality dimensions on pages 6 and 7, lines 144-162.

4. Avoid evaluative words like “good” without defining criteria. Comment accepted. Judgement criteria is a preset criteria developed based on previous evidence and by agreement with stakeholders and weight was given for each indicator by considering their relative importance in the evaluation, and presented on page 7, line 167-169.

5. Conceptual clarity of definitions of data quality and information use are provided but not clearly linked to the study framework. Thank you. We have introduced the definition of data quality and information use as a whole since HIV/AIDS program is key component of health programs. In addition, we have highlighted HIV/AIDS program data quality on page 3, line 63-65.

6. Reorganize the problem statement into Local evidence gap and study rationale and clearly state why Jimma town is an important setting. We presented the problem statement from broader to specific (global to study area), and presented that there is no published study conducted on HMIS or HIV AIDS program data quality and information use in Jimma town on page 4, line 74-90.

7. The term “facility based case study design” is methodologically ambiguous. So, clearly define the design, e.g.: “A facility-based cross-sectional evaluation using mixed quantitative and qualitative methods.” Comment accepted. We have modified the study design to “facility-based cross-sectional evaluation using mixed quantitative and qualitative methods” on page 2 line 29, and page 4, line 94.

8. Justification for selecting 234 records is mathematically cluttered and hard to follow. The sample size was determined by using single population proportion formula taking P 0.825 for overall average data quality of HMIS which was 82.5% from study conducted in Southern Ethiopia. Then, correction formula was used to get a sample size of 234 since the total population is less than 10,000 as presented on page 5, line 104-115.

9. Inclusion of qualitative sample lacks clear rationale. For qualitative study, purposive sampling technique was used in order to get information reach individuals and sample size was determined by saturation of idea as stated 0n page 5, line 114-116. Accordingly, the purpose was key informants level of stake i.e being ART providers, ART focal person, M&E officer, ART coordinator and data clerk.

10. Tools are mentioned but not sufficiently described. And it is unclear how qualitative data informed quantitative findings. Qualitative and quantitative data are collected simultaneously, and qualitative results were narrated by triangulating with quantitative findings, page 6, lines 131-136

11. Analytical methods are largely descriptive, yet the manuscript uses evaluative judgments (“good”). Mixed method was used in this evaluation, and a preset criteria judgment criteria which was developed by agreement with stakeholders was used and presented on page 7, line 168-170.

12. Weighting system and judgment parameters are not clearly justified. Comment accepted. We have included how judgement criteria were developed and weight was given for each indicator and presented on pages 6 and7, lines 145-170.

13. Operational Definitions: Definitions are long, repetitive, and sometimes circular. Comment accepted. We have modified the operational definitions and presented in informative way by including expected national standards for each data quality dimensions on pages 6 and 7 , lines 143-170.

14. Results section includes interpretation that should be in Discussion.

In our evaluation qualitative results were presented by triangulating with quantitative findings to provide detail explanation for the quantitative results. Hence, we used introductory statement to present the actual responses of the key informants which in some ways resemble interpretation.

15. Some percentages reported in text do not always align clearly with tables. Comment accepted. Thank you so much for your careful review. The percentage in the narration (80%) indicates the overall availability of basic infrastructures in Jimma town public health facilities while the result in the table shows the availability of basic infrastructures in each health facilities. So, we have paraphrased the statement to make it informative on page 9, line 203&204.

16. Use of terms such as overall, facility level, program level interchangeably. Comment accepted. We have included your comments and removed inappropriate statement on page 10, line 225&226.

17. Discussion largely repeats results. We briefly narrated the main results under discussion section to compare our findings with different literatures and provide explanation.

18. Limited critical reflection on methodological limitations. Thank you. We have elaborated methodological limitations, and how the limitation could affect the interpretation on page 17, line 366 -370.

19. Causal language is occasionally used despite descriptive design. We provided the possible explanation for different level of achievement as compared to literature rather than cause effect relationship.

20. Strengthen linkage to Ethiopian HMIS policy and HIV program goals. Thank you. We discussed our findings with the expected national standards and presented by justifying with Ethiopian HMIS policy and HIV program goals on page 16, line 349-352.

21. The limitations are acknowledged but underdeveloped. Please, clarify how these limitations affect interpretation. Comment accepted. We have elaborated how the limitation could affect the interpretation on page 17, line 366-370.

22. Conclusions align with objectives. However, some conclusions repeat numerical findings and recommendations are generic. I suggest you to tailor recommendations to specific bodies. Comment accepted. We have incorporated your comments and forwarded the recommendation for specific bodies on page 17, line 378-380.

Response to editors queries

23. Please clarify the source of funding (financial or material support) for your study. State what role the funders took in the study. Comment accepted. The source of funding (support) and their role was stated on page 19, line 412-414.

24. We note that you have provided funding information that is not currently declared in your Funding Statement. However, funding information should not appear in the Acknowledgments section or other areas of your manuscript. We have removed from the acknowledgement section of the manuscript, and included the amended statements within the cover letter.

25. Please amend your authorship list in your manuscript file to include the full name of author Tilahun Fufa Debela. Thank you. We have included it.

26. Your full ethics statement should only appear in the Methods section of your manuscript. If your ethics statement is written in any section besides the Methods, please delete it from any other section. Comment accepted. We have deleted from other section of the manuscript other than method section.

27. Please note that there are currently two tables labeled as "Table 1" on pages 9 and 13. Please renumber your tables and update the corresponding citations in the text to ensure they follow a sequential order. Thank you. We have renumbered it.

28. Please remove or anonymize all personal information (specific dates, health facility names, unique ART numbers), ensure that the data shared are in accordance with participant consent, and re-upload a fully anonymized data set. Please note that spreadsheet columns with personal information must be removed and not hidden as all hidden columns will appear in the published file. We didn’t presented personal information like name of client or unique ART numbers in the manuscript, rather we assessed the completeness of patient card and registration by checking whether demographic information like name, age, unique ART numbers are completely filled or not.

Attachment Submitted filename: Reponse to Reviewers.pdf

# Editor report: Decision Letter 1

**DOI:** 10.1371/journal.pone.0357022.r003

**Authors:** Worku Asferie

11 Aug 2026

Evaluation of Health Management Information System Data Quality and Information Use for HIV/AIDS Programs in Public Health Facilities of Jimma town, Southwest Ethiopia

PONE-D-25-65187R1

Dear Dr. Berhane Megerssa Ereso,

We’re pleased to inform you that your manuscript has been judged scientifically suitable for publication and will be formally accepted for publication once it meets all outstanding technical requirements.

Within one week, you’ll receive an e-mail detailing the required amendments. When these have been addressed, you’ll receive a formal acceptance letter and your manuscript will be scheduled for publication.

An invoice will be generated when your article is formally accepted. Please note, if your institution has a publishing partnership with PLOS and your article meets the relevant criteria, all or part of your publication costs will be covered. Please make sure your user information is up-to-date by logging into Editorial Manager at Editorial Manager® and clicking the ‘Update My Information' link at the top of the page. For questions related to billing, please contact billing support.

If your institution or institutions have a press office, please notify them about your upcoming paper to help maximize its impact. If they’ll be preparing press materials, please inform our press team as soon as possible -- no later than 48 hours after receiving the formal acceptance. Your manuscript will remain under strict press embargo until 2 pm Eastern Time on the date of publication. For more information, please contact onepress@plos.org.

Kind regards,

Worku Necho Asferie, MSc

Academic Editor

PLOS One

Additional Editor Comments (optional):

• Operational definition: most of the operational definitions need reference especially, Judgment criteria is required reference.

• Please clearly state when the patient record is excluded from this study

• How can you assure the data quality for this study?

• Strictly correct any grammatical and spelling error

Reviewers' comments:

# Editor report: Acceptance letter

**DOI:** 10.1371/journal.pone.0357022.r004

**Authors:** Worku Asferie

PONE-D-25-65187R1

PLOS One

Dear Dr. Ereso,

I'm pleased to inform you that your manuscript has been deemed suitable for publication in PLOS One. Congratulations! Your manuscript is now being handed over to our production team.

At this stage, our production department will prepare your paper for publication. This includes ensuring the following:

* All references, tables, and figures are properly cited

* All relevant supporting information is included in the manuscript submission,

* There are no issues that prevent the paper from being properly typeset

You will receive further instructions from the production team, including instructions on how to review your proof when it is ready. Please keep in mind that we are working through a large volume of accepted articles, so please give us a few days to review your paper and let you know the next and final steps.

Lastly, if your institution or institutions have a press office, please let them know about your upcoming paper now to help maximize its impact. If they'll be preparing press materials, please inform our press team within the next 48 hours. Your manuscript will remain under strict press embargo until 2 pm Eastern Time on the date of publication. For more information, please contact onepress@plos.org.

You will receive an invoice from PLOS for your publication fee after your manuscript has reached the completed accept phase. If you receive an email requesting payment before acceptance or for any other service, this may be a phishing scheme. Learn how to identify phishing emails and protect your accounts at https://explore.plos.org/phishing.

If we can help with anything else, please email us at customercare@plos.org.

Thank you for submitting your work to PLOS One and supporting open access.

Kind regards,

PLOS One Editorial Office Staff

on behalf of

Assistant Professor Worku Necho Asferie

Academic Editor

PLOS One
